# From Bilinear Regression to Inductive Matrix Completion: A Quasi-Bayesian Analysis

**DOI:** 10.3390/e25020333

**Published:** 2023-02-11

**Authors:** The Tien Mai

**Affiliations:** Department of Mathematical Sciences, Norwegian University of Science and Technology, 7034 Trondheim, Norway; the.t.mai@ntnu.no

**Keywords:** bilinear regression, matrix completion, low-rank model, PAC-Bayesian bound, Langevin Monte Carlo

## Abstract

In this paper, we study the problem of bilinear regression, a type of statistical modeling that deals with multiple variables and multiple responses. One of the main difficulties that arise in this problem is the presence of missing data in the response matrix, a problem known as inductive matrix completion. To address these issues, we propose a novel approach that combines elements of Bayesian statistics with a quasi-likelihood method. Our proposed method starts by addressing the problem of bilinear regression using a quasi-Bayesian approach. The quasi-likelihood method that we employ in this step allows us to handle the complex relationships between the variables in a more robust way. Next, we adapt our approach to the context of inductive matrix completion. We make use of a low-rankness assumption and leverage the powerful PAC-Bayes bound technique to provide statistical properties for our proposed estimators and for the quasi-posteriors. To compute the estimators, we propose a Langevin Monte Carlo method to obtain approximate solutions to the problem of inductive matrix completion in a computationally efficient manner. To demonstrate the effectiveness of our proposed methods, we conduct a series of numerical studies. These studies allow us to evaluate the performance of our estimators under different conditions and provide a clear illustration of the strengths and limitations of our approach.

## 1. Introduction

In this paper, we investigate the bilinear regression model, a statistical method that assumes a linear relationship between a set of multiple response variables and two sets of covariates. This model, also known as the growth curve model or generalized multivariate analysis model, is commonly used for analyzing longitudinal data, as shown in previous studies such as [1,2,3,4,5,6]. However, these studies only cover the scenario in which the response matrix is fully observed.

Recently, the bilinear regression model with incomplete response has been introduced and studied as the so-called inductive matrix completion, which is a generalization of the matrix completion problem [7,8]. This problem has attracted significant attention in various fields, such as drug repositioning [9], collaborative filtering [10], and genomics [7]. Inductive matrix completion is a challenging problem that arises when some of the entries in the response matrix are missing, which makes it difficult to infer the underlying relationship between the variables.

In this work, we explore the problem of bilinear regression and inductive matrix completion under a low-rank constraint on the coefficient matrix. Most existing approaches for these problems are frequentist methods, such as maximum likelihood estimation [2] or penalized optimization [7]. These methods are effective in providing point estimates for the parameters of the model but lack the ability to provide a full probabilistic characterization of the uncertainty. Recently, Bayesian approaches have been considered for these problems. For example, the paper [11] proposed a Bayesian approach for bilinear regression, and a Bayesian method was proposed for inductive matrix completion in the work [9]. However, unlike frequentist approaches, the statistical properties of the Bayesian approach for these models have not been fully explored yet.

The aim of this paper is to address an existing gap in the understanding of the bilinear regression and inductive matrix completion problems. To achieve this goal, we propose a novel approach that combines elements of Bayesian statistics with a quasi-likelihood method. Specifically, we start by addressing the problem of bilinear regression using a quasi-Bayesian approach, where a quasi-likelihood is employed. We then generalize this approach to the problem of inductive matrix completion. To ensure that our method is adaptive to the rank of the coefficient matrix, we use a spectral scaled Student prior distribution, which allows us to prove that the posterior mean satisfies a tight oracle inequality. This result demonstrates that our method is able to accurately estimate the parameters of the model, even when the rank of the coefficient matrix is unknown. Additionally, we also prove the contraction properties of the posteriors, which further enhances the performance of our method.

The proposed method in this paper, the quasi-Bayesian approach, is an extension of the traditional Bayesian approach and is becoming increasingly popular in statistics and machine learning as a technique for generalized Bayesian inference, as noted in studies such as [12,13,14]. This approach allows for more flexibility in the modeling assumptions by replacing the likelihood function with a more general notion of risk or quasi-likelihood.

To provide theoretical guarantees for our proposed quasi-posteriors, we make use of the PAC-Bayesian technique [15,16,17]. This technique provides bounds on the generalization error of a learned estimator, and has been widely used in the literature as described in recent reviews and introductions, such as [18,19]. The PAC-Bayes bounds have been successfully applied in the context of matrix estimation problems as shown in studies such as [20,21,22,23]. Interestingly, by using the PAC-Bayesian technique for inductive matrix completion, we do not need to make any assumptions about the distribution of the missing entries in the response matrix. This is in contrast with previous works on matrix completion, such as [24,25,26,27,28,29], which typically require assumptions about the missing data. This makes our method more versatile and applicable to a wider range of problems.

The proposed method in this paper makes use of a spectral scaled Student prior, which is a specific choice of prior distribution. This choice is motivated by recent works in which it has been shown to lead to optimal rates in a variety of problems, including high-dimensional regression [30], image denoising [31] and reduced rank regression [23]. Although this prior is not conjugate to our problems, it allows for the convenient implementation of gradient-based sampling methods, which makes it computationally efficient.

To compute the proposed estimators and sample from the quasi-posterior, we employ a Langevin Monte Carlo (LMC) method. This method is a widely used algorithm for approximating complex distributions and allows for efficient computation of the proposed estimators. The LMC method allows us to obtain approximate solutions to the problem of bilinear regression and inductive matrix completion in a computationally efficient manner.

Furthermore, we use numerical studies to demonstrate the effectiveness of our proposed methods. These studies enable us to evaluate the performance of our estimators in various scenarios and provide insight into the capabilities and limitations of our approach. By conducting a thorough evaluation, we can gain a better understanding of how our method performs in different settings and make any necessary adjustments to improve its performance. We also compare our method with the ordinary least squared method. This comparison allows us to demonstrate the superiority of our method over the traditional approach in certain scenarios, such as when the response matrix contains missing data. Overall, the numerical studies serve as a valuable tool for assessing the effectiveness of our proposed method and provide a clear illustration of its strengths and weaknesses, as well as its improvement over the traditional method.

The remainder of this paper is organized as follows: In Section 2, we present the problem of bilinear regression, and introduce the low-rank promoting prior distribution that we use to address this problem. In Section 3, we extend our approach to the problem of inductive matrix completion. In Section 4, we discuss the Langevin Monte Carlo method used for the computation of the estimators, and present numerical studies to demonstrate the effectiveness of our proposed methods. Finally, we conclude our work and provide a summary of our findings in Section 5. The technical proofs are provided in Appendix A for the interested readers.

**Notation** **1.** *Let $R^{n_{1}\times n_{2}}$ denote the set of $n_{1}\times n_{2}$ matrices with real elements. Let $A^{⊺}\in R^{n_{2}\times n_{1}}$ denote the transpose of A. For any $A\in R^{n_{1}\times n_{2}}$ and $I=\left(i,j\right)\in \left\{1,\ldots ,n_{1}\right\}\times \left\{1,\ldots ,n_{2}\right\}$, we denote by $A_{I}=A_{\left(i,j\right)}=A_{i,j}$ the i-th row and j-th column elements of A. The matrix in $R^{n_{1}\times n_{2}}$ with all entries equal to 0 is denoted by $0_{n_{1}\times n_{2}}$. For a matrix $B\in R^{n_{1}\times n_{1}}$, we let Tr(B) denote its trace. The identity matrix in $R^{n_{1}\times n_{1}}$ is denoted by $I_{n_{1}}$. For $A\in R^{n_{1}\times n_{2}}$, we define its sup-norm ${∥A∥}_{\infty }=\max_{i,j}\left|A_{i,j}\right|$; its Frobenius norm ${∥A∥}_{F}$ is defined by ${∥A∥}_{F}^{2}=\mathrm{Tr}\left(A^{⊺}A\right)=\sum_{i,j}A_{i,j}^{2}$ and rank(A) its rank.*

## 2. Bilinear Linear Regression

### 2.1. Model

Let $Y\in R^{n\times q}$ consist of *n* independent response vectors, $X\in R^{n\times p}$ be a given between-individuals design matrix and $Z\in R^{k\times q}$ be a known within-individuals design matrix. Consider the bilinear regression model as follows:

(1) $$Y=XM^{*}Z+E,$$

where $M^{*}\in R^{p\times k}$ is the unknown parameter matrix. The random noise matrix *E* is assumed to have zero mean, E(E)=0. The main assumption here is the low-rank restriction on the model parameter that $\mathrm{rank}\left(M^{*}\right)<\min\left(p,k\right)$.

The model presented in Equation (1) is a bilinear regression model, which can be seen as a generalization of the reduced rank regression problem. In the case where k=q and $Z=I_{q}$, the model simplifies to the traditional reduced rank regression problem, which has been well-studied in the literature, such as [32,33]. However, in this paper, we consider the more general case where the matrix *Z* contains additional explanatory variables.

The low-rank assumption in this model can be interpreted as indicating the presence of a latent process that affects the response data, not only through the “between-individuals” structure of the model but also through the “within-individuals” structure. This model is often referred to as the growth curve model or the generalized multivariate analysis model (GMANOVA) and has been studied in depth in the literature, such as [2].

**Assumption** **1.** There is a known constant C<+∞ such that $∥XM^{*}{Z∥}_{\infty }\leq C.$

From Assumption 1, it is not reliable to return predictions XMZ with entries that are outside of interval [−C,C]. However, for computational reasons, it is extremely convenient to employ an unbounded prior for *M*. Therefore, we propose to use unbounded distributions for *M* but to use, as a predictor, a truncated version of XMZ rather than *M* itself. For a matrix *A*, let

$$\Pi_{C}\left(A\right)=\arg\min_{{∥B∥}_{\infty }\leq C}{∥A-B∥}_{F}$$

be the orthogonal projection of *A* on matrices with entries bounded by *C*. Note that *B* is simply obtained by replacing entries of *A* larger than *C* by *C*, and entries smaller than −C by −C.

For a matrix $M\in R^{p\times k}$, we denote by r(M) the empirical risk of *M* as

$$r\left(M\right)=\frac{1}{nq}{∥Y-\Pi_{C}\left(XMZ\right)∥}_{F}^{2}$$

and its expectation is denoted by

$$R\left(M\right)=E\left[r(M)\right]=E\left[{\left(Y_{11}-{\left(\Pi_{C}\left(XMZ\right)\right)}_{{}_{11}}\right)}^{2}\right].$$

The focus of our work in this paper is on the predictive aspects of the model, that is, a matrix *M* predicts almost as well as $M^{*}$ if $R\left(M\right)-R\left(M^{*}\right)$ is small. Under the assumption that $E_{ij}$ has a finite variance, using the Pythagorean theorem, we have

(2) $$R\left(M\right)-R\left(M^{*}\right)=\frac{1}{nq}{∥\Pi_{C}\left(XMZ\right)-XM^{*}Z∥}_{F}^{2}$$

for any *M*, which means that our results can also be interpreted in terms of the Frobenius norm.

Let π be a prior distribution on $R^{p\times k}$ (see Section 2.2). For any λ>0, we define the quasi-posterior

$${\hat{\rho }}_{\lambda }\left(dM\right)\propto \exp\left(-\lambda r\left(M\right)\right)\pi \left(dM\right).$$

It is worth noting that for a specific choice of $\lambda =nq/(2\sigma^{2})$, the posterior distribution obtained corresponds to the case where the noise term $E_{ij}$ is assumed to be Gaussian distributed with a mean of 0 and a variance of $\sigma^{2}$. However, our theoretical results hold under a more general class of noise distributions. It is known that a small enough λ is sufficient when the model is misspecified [14]. Additionally, even in the case of Gaussian noise, in high-dimensional settings, a smaller value of λ than $n/(2\sigma^{2})$ leads to better adaptation properties [30,34]. The precise choice of λ in our method will be further discussed below.

We consider the following posterior mean of XMZ, given by

(3) $${\hat{XMZ}}_{\lambda }=\int \Pi_{C}\left(XMZ\right){\hat{\rho }}_{\lambda }\left(dM\right).$$

It is worth noting that from the simulation experiments, it is observed that using reasonable values for *C*, the Monte Carlo algorithm never samples matrices *M* such that $\Pi_{C}\left(XMZ\right)\neq XMZ$. In other words, the boundedness constraint has very little impact on practice, and it is mainly necessary for technical proofs. If one is interested in obtaining an estimator of $M^{*}$ instead of an estimator of $XM^{*}Z$, when $X^{⊺}X$ and $ZZ^{⊺}$ are invertible, one can consider the estimator ${\hat{M}}_{\lambda }={\left(X^{⊺}X\right)}^{-1}X^{⊺}{\hat{XMZ}}_{\lambda }Z^{⊺}{\left(ZZ^{⊺}\right)}^{-1}$ and note that $X{\hat{M}}_{\lambda }Z={\hat{XMZ}}_{\lambda }$. This estimator can be used to obtain the estimator of $M^{*}$ in case the inverses of $X^{⊺}X$ and $ZZ^{⊺}$ are computationally feasible.

The quasi-posterior distribution investigated in this paper is often referred to as the “Gibbs posterior” in the PAC-Bayes approach. This terminology is used in the literature such as [17,18,19,35,36]. Additionally, the estimator ${\hat{M}}_{\lambda }$ is sometimes referred to as the Gibbs estimator or the exponentially weighted aggregate (EWA) in the literature, such as [34,37,38].

### 2.2. Prior Specification

We consider, in this paper, the following spectral scaled Student prior distribution, with parameter τ>0:

(4) $$\begin{array}{c} \pi \left(M\right)\propto \det{\left(\tau^{2}I_{m}+MM^{⊺}\right)}^{-(p+m+2)/2}. \end{array}$$

This prior distribution is designed to promote low-rankness by placing more probability mass on matrices with smaller singular values, which leads to sparse solutions. This prior has a similar form to the one used in related works such as [39,40], and it is known to lead to good performance in different problems, such as high-dimensional regression and image denoising. It can be verified that

$$\pi \left(M\right)\propto \prod_{j=1}^{m}{\left(\tau^{2}+s_{j}{\left(M\right)}^{2}\right)}^{-(p+m+2)/2},$$

where $s_{j}\left(M\right)$ denotes the *j*th largest singular value of *M*. The above expression is a scaled Student distribution evaluated at $s_{j}\left(M\right)$, which can be seen as a way to approximate sparsity on $s_{j}\left(M\right)$ [30]. The log-sum function $\sum_{j=1}^{m}\log\left(\tau^{2}+s_{j}{\left(M\right)}^{2}\right)$ used by [39,40] is also known to enforce approximate sparsity on the singular values of $s_{j}\left(M\right)$. This means that under this prior, most of the $s_{j}\left(M\right)$ are close to 0, which implies that *M* is well approximated by a low-rank matrix. Therefore, it has the ability to promote the low-rankness of *M*.

As previously stated, this prior is not conjugate to the problem at hand. However, it is particularly convenient to implement gradient-based sampling algorithms, such as the Langevin Monte Carlo method, which will be discussed in more detail in Section 4. This is because the gradient of the log-posterior can be computed efficiently, and it allows for an efficient implementation of the LMC algorithm.

### 2.3. Theoretical Results

We assume the sub-exponential distribution assumption on the noise.

**Assumption** **2.** *The entries $E_{i,j}$ of E are independent. There exist two known constants σ>0 and ξ>0 such that*

$$\forall k\geq 2,\;E(|E_{i,j}{|}^{k})\leq \sigma^{2}k!\xi^{k-2}/2.$$

Let us put

$$C_{1}=8\left(\sigma^{2}+C^{2}\right);\;C_{2}=64C\max\left(\xi ,C\right);\;\tau^{*}=\sqrt{C_{1}\left(k+p\right)/{(nkq∥X∥}_{F}^{2}{∥Z∥}_{F}^{2})}.$$

The statistical properties of mean estimator are given in the following theorem, where we propose a non-asymptotic analysis for our mean estimator.

**Theorem** **1.** Let Assumptions 1 and 2 be satisfied. Fix the parameter $\tau =\tau^{*}$ in the prior. Fix δ>0 and define $\lambda^{*}:=nq\min\left(1/\left(2C_{2}\right),\delta /\left[C_{1}\left(1+\delta \right)\right]\right)$. Then, for any ε∈(0,1), we have, with probability at least 1−ε on the sample,

$$\begin{array}{c} {∥{\hat{XMZ}}_{\lambda^{*}}-XM^{*}Z∥}_{F}^{2}\leq \underset{0\leq r\leq pk}{\inf}\underset{\begin{array}{c} \begin{array}{c} \bar{M}\in R^{p\times k}\\ \mathrm{rank}(\bar{M})\leq r \end{array} \end{array}}{\inf}\{\left(1+\delta \right){∥X\bar{M}Z-XM^{*}Z∥}_{F}^{2}\;+\\ \frac{C_{1}{\left(1+\delta \right)}^{2}}{\delta }\left[4r\left(k+p+2\right)\log\left(1+\frac{{∥X∥}_{F}{∥Z∥}_{F}{∥\bar{M}∥}_{F}}{\sqrt{C_{1}}}\sqrt{\frac{nkq}{r(k+p)}}\right)+k+p+2\log\frac{2}{\varepsilon }\right]\}. \end{array}$$

The choice of $\lambda =\lambda^{*}$ is determined by optimizing an upper bound on the risk *R* (as shown in the proof of this theorem). However, it is important to note that this choice may not necessarily be the best choice in practice, even though it gives a good estimate of the order of magnitude for λ. To ensure optimal performance, the user can use cross-validation to properly adjust the temperature parameter. Additionally, it is worth noting that $\mathrm{rank}(\bar{M})\neq 0$ is not a requirement in the above formula. If $\mathrm{rank}(\bar{M})=0$, then $\bar{M}=0$ and we interpret 0log(1+0/0) as 0.

The proof of this theorem is based on the PAC-Bayes theory, and it is provided in the Appendix A. By taking $\bar{M}=M^{*}$, we can obtain an upper bound on the infimum, leading to the following result.

**Corollary** **1.** Under the same assumptions and the same $\tau ,\lambda^{*}$ as in Theorem 1, let $r^{*}=\mathrm{rank}\left(M^{*}\right)$. Then, for any ε∈(0,1), we have, with probability at least 1−ε on the sample,

$$\begin{array}{c} {∥{\hat{XMZ}}_{\lambda^{*}}-XM^{*}Z∥}_{F}^{2}\leq \\ \frac{C_{1}{\left(1+\delta \right)}^{2}}{\delta }\left[4r^{*}\left(k+p+2\right)\log\left(1+\frac{{∥X∥}_{F}{∥Z∥}_{F}{∥M^{*}∥}_{F}}{\sqrt{C_{1}}}\sqrt{\frac{nkq}{r(k+p)}}\right)+k+p+2\log\frac{2}{\varepsilon }\right]. \end{array}$$

Theorem 1 provides an understanding of the statistical properties of the posterior mean. However, it is also important to understand the contraction properties of the quasi-posterior distribution. In the following theorem, we aim to provide a result that demonstrates this aspect of the proposed method.

**Theorem** **2.** *Under the assumptions for Theorem 1, let $\varepsilon_{n}$ be any sequence in (0,1) such that $\varepsilon_{n}\to 0$ when n→∞. Define*

$$\begin{array}{c} M_{n}=\{M\in R^{p\times k}:{∥{\hat{XMZ}}_{\lambda^{*}}-XM^{*}Z∥}_{F}^{2}\leq \underset{0\leq r\leq pk}{\inf}\underset{\begin{array}{c} \begin{array}{c} \bar{M}\in R^{p\times k}\\ \mathrm{rank}(\bar{M})\leq r \end{array} \end{array}}{\inf}\{\left(1+\delta \right){∥X\bar{M}Z-XM^{*}Z∥}_{F}^{2}\;+\\ \frac{C_{1}{\left(1+\delta \right)}^{2}}{\delta }\left[4r\left(k+p+2\right)\log\left(1+\frac{{∥X∥}_{F}{∥Z∥}_{F}{∥\bar{M}∥}_{F}}{\sqrt{C_{1}}}\sqrt{\frac{nkq}{r(k+p)}}\right)+k+p+2\log\frac{2}{\varepsilon_{n}}\right]\}. \end{array}$$

*Then,*

$$E\left[P_{M∼{\hat{\rho }}_{\lambda }}\left(M\in M_{n}\right)\right]\geq 1-\varepsilon_{n}\underset{n\to \infty }{\to }1.$$

The proof of this theorem is provided in Appendix A.

## 3. Inductive Matrix Completion

### 3.1. Model and Method

In the context of inductive matrix completion, given two side information matrices *X* and *Z*, we assume that only a random subset Ω of the entries of *Y* in model (1) is observed. More precisely, we assume that we observe *m* independent and identically random pairs $\left(I_{1},Y_{1}\right),\ldots ,\left(I_{m},Y_{m}\right)$ given by

(5) $$Y_{i}={\left(XM^{*}Z\right)}_{I_{i}}+E_{i},\;i=1,\ldots ,m$$

where $M^{*}\in R^{p\times k}$ is the unknown parameter matrix expected to be low-rank and observation sample size is assumed that m<nq. The noise variables $E_{i}$ are assumed to be independent with $E(E_{i})=0.$ The variables $I_{i}$ are independent and identical copies of a random variable I having distribution Π on the set {1,…,n}×{1,…,q}, we denote $\Pi_{x,y}:=\Pi \left(I=\left(x,y\right)\right)$.

Our goal in this paper is to investigate the problem of bilinear regression and also address the case where the response matrix contains missing data, a problem known as inductive matrix completion. In particular, when p=n,k=q and $X=I_{n},Z=I_{q}$ are the identity matrices, the problem reduces to the traditional matrix completion problem, which has been well studied in the literature [26]. Similarly, when k=q and $Z=I_{q}$ is the identity matrix, the problem becomes the reduced rank regression problem with incomplete response, which has also been studied in recent works, such as [23,41]. However, in the context of inductive matrix completion, we focus on the more general case where *X* and *Z* contain additional explanatory variables; in other words, we consider the side information from the *n* users and the *q* items in our model [8].

It has been acknowledged that there are two different ways to model the observed values of *Y*, either by including or excluding the possibility of observing the same entry multiple times. Previous studies have examined both of these methods, such as the examination of matrix completion without replacement in [25] and with replacement in [26]. Both methods have practical uses and use similar techniques for estimation. This particular study focuses on the scenario where the variables $I_{i}$ are independently and identically distributed, meaning that it is possible to observe the same entry multiple times. Additionally, it is important to note that, according to the findings presented in Section 6 of [42], the results of this study can also be applied to the scenario of sampling without replacement, as long as the sampling is performed uniformly and there is no observation noise.

We are now adapting the quasi-Bayesian approach for bilinear regression in Section 2 to the context of inductive matrix completion. For a probability distribution *P* on $\left\{1,\ldots ,n_{1}\right\}\times \left\{1,\ldots ,n_{2}\right\}$, we generalize the Frobenius norm by ${∥A∥}_{F,P}^{2}=\sum_{i,j}P\left[\left(i,j\right)\right]A_{i,j}^{2}$; note that when *P* is the uniform distribution, then ${∥A∥}_{F,P}^{2}={∥A∥}_{F}^{2}/\left(n_{1}n_{2}\right)$.

For a matrix $M\in R^{p\times k}$, we denote the empirical risk of *M*, $r^{\prime }\left(M\right)$, and its expected risk $R^{\prime }\left(M\right)$ respectively as

$$r^{\prime }\left(M\right)=\frac{1}{m}\sum_{i=1}^{m}{\left(Y_{i}-{\left(\Pi_{C}\left(XMZ\right)\right)}_{I_{i}}\right)}^{2},$$

$$R^{\prime }\left(M\right)=E\left[r^{\prime }\left(M\right)\right]=E\left[{\left(Y_{1}-{\left(\Pi_{C}\left(XMZ\right)\right)}_{I_{1}}\right)}^{2}\right].$$

As in Section 2, we will focus on the predictive aspects of the model, that is, a matrix *M* predicts almost as well as $M^{*}$ if $R^{\prime }\left(M\right)-R^{\prime }\left(M^{*}\right)$ is small. Under the assumption that $E_{i}$ has a finite variance, based on the Pythagorean theorem, we have

(6) $$R^{\prime }\left(M\right)-R^{\prime }\left(M^{*}\right)={∥\Pi_{C}\left(XMZ\right)-XM^{*}Z∥}_{F,\Pi }^{2}$$

for any *M*, which means that our results can also be interpreted in terms of an estimation of $M^{*}$ with respect to a generalized Frobenius norm.

Here, the prior π is the low-rank inducing prior specified in the Section 2.2 above. For any λ>0, we define the quasi-posterior

$${\hat{\rho }}_{\lambda }^{\prime }\left(dM\right)\propto \exp\left(-\lambda r^{\prime }\left(M\right)\right)\pi \left(dM\right).$$

We will actually specify our choice of λ below.

The truncated posterior mean of XMZ is given by

(7) $${\hat{XMZ}}_{\lambda }=\int \Pi_{C}\left(XM\right){\hat{\rho }}_{\lambda }^{\prime }\left(dM\right).$$

Here, for the same technical reasons as in the context of bilinear regression, this truncation has a very little impact in practice for reasonable values of *C*.

### 3.2. Theoretical Results

In this section, we derive the statistical properties of the posterior ${\hat{\rho }}_{\lambda }^{\prime }$ and the mean estimator ${\hat{XMZ}}_{\lambda }$ for the context of inductive matrix completion. Let us first state our assumptions on this model.

**Assumption** **3.** *The noise variables $E_{1},\ldots ,E_{m}$ are independent of $I_{1},\ldots ,I_{m}$. There exist two known constants $\sigma^{\prime }>0$ and $\xi^{\prime }>0$ such that*

$$\forall k\geq 2,\;E(|E_{i}{|}^{k})\leq \sigma^{\prime 2}k!\xi^{\prime k-2}/2.$$

Assumptions 1 and 3 are both standard; they have been used in [41] for theoretical analysis of reduced rank regression and in [26] for trace regression and matrix completion.

Let us put

$$C_{1}^{\prime }=8\left(\sigma^{\prime 2}+C^{2}\right);\;C_{2}^{\prime }=64C\max\left(\xi^{\prime },C\right);\;\tau^{*}=\sqrt{C_{1}^{\prime }\left(k+p\right)/{(mkp∥X∥}_{F}^{2}{∥Z∥}_{F}^{2})}.$$

**Theorem** **3.** Let Assumptions 1 and 3 be satisfied. Fix the parameter $\tau =\tau^{*}$ in the prior. Fix δ>0 and define $\lambda^{\prime *}:=m\min\left(1/\left(2C_{2}^{\prime }\right),\delta /\left[C_{1}^{\prime }\left(1+\delta \right)\right]\right)$. Then, for any ε∈(0,1), we have, with probability at least 1−ε on the sample,

$$\begin{array}{c} {∥{\hat{XMZ}}_{\lambda^{\prime *}}-XM^{*}Z∥}_{F,\Pi }^{2}\leq \underset{0\leq r\leq pk}{\inf}\underset{\begin{array}{c} \begin{array}{c} \bar{M}\in R^{p\times k}\\ \mathrm{rank}(\bar{M})\leq r \end{array} \end{array}}{\inf}\{\left(1+\delta \right){∥X\bar{M}Z-XM^{*}Z∥}_{F,\Pi }^{2}+\\ \frac{C_{1}^{\prime }{\left(1+\delta \right)}^{2}}{\delta }\frac{\left(4r\left(k+p+2\right)\log\left(1+\frac{{∥X∥}_{F}{∥Z∥}_{F}{∥\bar{M}∥}_{F}}{\sqrt{C_{1}}}\sqrt{\frac{mkp}{r(k+p)}}\right)+k+p+2\log\frac{2}{\varepsilon }\right)}{m}\}. \end{array}$$

Similar to the context of bilinear regression, the choices of $\lambda =\lambda^{*},\tau =\tau^{*}$ come from the optimization of an upper bound on the risk *R* (in the proof of this theorem). Therefore, these choices may not be necessarily the best choice in practice, even though it gives a good order of magnitude for tuning these parameters. The user could use cross-validation to properly tune them in practice. Note again that $\mathrm{rank}(\bar{M})\neq 0$ is not required in the above formula, if $\mathrm{rank}(\bar{M})=0$ then $\bar{M}=0$ and we interpret 0log(1+0/0) as 0. The proof of this theorem is provided in the Appendix A. In particular, we can upper bound the infimum on $\bar{M}$ by taking $\bar{M}=M^{*}$, which leads to the following result.

**Corollary** **2.** Under the assumptions that Theorem 3 holds, let $r^{*}=\mathrm{rank}\left(M^{*}\right)$. Put

$$R_{\delta ,m,p,k,r^{*},\varepsilon }:=\frac{C_{1}^{\prime }{\left(1+\delta \right)}^{2}}{\delta }\frac{\left(4r\left(k+p+2\right)\log\left(1+\frac{{∥X∥}_{F}{∥Z∥}_{F}{∥\bar{M}∥}_{F}}{\sqrt{C_{1}}}\sqrt{\frac{mkp}{r(k+p)}}\right)+k+p+2\log\frac{2}{\varepsilon }\right)}{m},$$

*then*

$$\begin{array}{c} {∥{\hat{XMZ}}_{\lambda^{\prime *}}-XM^{*}Z∥}_{F,\Pi }^{2}\leq R_{\delta ,m,p,k,r^{*},\varepsilon } \end{array}$$

*and in particular, if the sampling distribution* Π *is uniform,*

$$\begin{array}{c} \frac{∥{\hat{XMZ}}_{\lambda^{\prime *}}-XM^{*}{Z∥}_{F}^{2}}{nq}\leq R_{\delta ,m,p,k,r^{*},\varepsilon }. \end{array}$$

**Remark** **1.** *Up to a log-term, our error rate r(k+p)/m is similar to the best known up-to-date rate derived in [8].*

While Theorem 3 is about the finite sample convergence rate of the posterior mean, it is actually possible to prove that the quasi-posterior ${\hat{\rho }}_{\lambda }^{\prime }$ contracts around $M^{*}$ at the same rate.

**Theorem** **4.** Under the same assumptions for Theorem 3, and the same definition for τ and $\lambda^{*}$, let $\varepsilon_{m}$ be any sequence in (0,1) such that $\varepsilon_{m}\to 0$ when m→∞. Define

$$\begin{array}{c} \Omega_{m}=\{M\in R^{p\times k}:{∥\Pi_{C}\left(XMZ\right)-XM^{*}Z∥}_{F,\Pi }^{2}\leq \\ \underset{1\leq r\leq pk}{\inf}\underset{\begin{array}{c} \begin{array}{c} \bar{M}\in R^{p\times k}\\ \mathrm{rank}(\bar{M})\leq r \end{array} \end{array}}{\inf}[\left(1+\delta \right){∥X\bar{M}Z-XM^{*}Z∥}_{F,\Pi }^{2}+\\ \frac{C_{1}^{\prime }{\left(1+\delta \right)}^{2}}{\delta }\frac{\left(4r\left(k+p+2\right)\log\left(1+\frac{{∥X∥}_{F}{∥Z∥}_{F}{∥\bar{M}∥}_{F}}{\sqrt{C_{1}}}\sqrt{\frac{mkp}{r(k+p)}}\right)+k+p+2\log\frac{2}{\varepsilon_{m}}\right)}{m}]\}. \end{array}$$

*Then*

$$E\left[P_{M∼{\hat{\rho }}_{\lambda }^{\prime }}\left(M\in \Omega_{m}\right)\right]\geq 1-\varepsilon_{m}\underset{m\to \infty }{\to }1.$$

The proof of this theorem is provided in Appendix A.

## 4. Numerical Studies

### 4.1. Langevin Monte Carlo Implementation

In this section, we propose to sample from the (quasi) posterior, in Section 2 and Section 3, by a suitable version of the Langevin Monte Carlo (LMC) algorithm, a gradient-based sampling method. We propose to use a constant step-size unadjusted LMC algorithm; see [43] for more details. The algorithm is given by an initial matrix $M_{0}$ and the recursion

(8) $$M_{k+1}=M_{k}-h\nabla \log{\hat{\rho }}_{\lambda }\left(M_{k}\right)+\sqrt{2h}\;N_{k}\;k=0,1,\ldots$$

where h>0 is the step-size, ${\hat{\rho }}_{\lambda }$ is the (quasi) posterior and $N_{0},N_{1},\ldots$ are independent random matrices with independent and identical standard Gaussian entries. We provide a pseudo-code for LMC in Algorithm A1. For small values of the step-size *h*, the output of Algorithm A1, $\hat{M}$, is very close to the integral (3) of interest. However, for some *h* that may not be small enough, the sum can explode [44]. In such cases, we consider to include a Metropolis–Hastings correction in the algorithm. Another possible choice is to take a smaller *h* and restart the algorithm; although it slows down the algorithm, we keep some control over its time of execution. On the other hand, the Metropolis–Hastings approach ensures the convergence to the desired distribution; however, the algorithm is greatly slowed down because of an additional acceptance/rejection step at each iteration.

Next, we propose a Metropolis–Hasting correction to the LMC algorithm. It guarantees the convergence to the (quasi) posterior, and it also provides a useful way for choosing *h*. More precisely, we consider the update rule in (8) as a proposal for a new candidate:

(9) $$\begin{array}{c} {\tilde{M}}_{k+1}=M_{k}-h\nabla \log{\hat{\rho }}_{\lambda }\left(M_{k}\right)+\sqrt{2h}\;N_{k},\;k=0,1,\ldots , \end{array}$$

Note that the matrix ${\tilde{M}}_{k+1}$ is normally distributed with mean $M_{k}-h\nabla \log{\hat{\rho }}_{\lambda }\left(M_{k}\right)$ and the covariance matrices equal to 2h times the identity matrices. This proposal is then accepted or rejected according to the Metropolis–Hastings algorithm, where the proposal is accepted with probability:

(10) $$A_{MALA}:=\min\left\{1,\frac{{\hat{\rho }}_{\lambda }\left({\tilde{M}}_{k+1}\right)q\left(M_{k}|{\tilde{M}}_{k+1}\right)}{{\hat{\rho }}_{\lambda }\left(M_{k}\right)q\left({\tilde{M}}_{k+1}|M_{k}\right)}\right\},$$

where

$$q\left(x^{\prime }|x\right)\propto \exp\left(-\frac{1}{4h}{∥x^{\prime }-x+h\nabla \log{\hat{\rho }}_{\lambda }\left(x\right)∥}_{F}^{2}\right)$$

is the transition probability density from *x* to $x^{\prime }$. The details of the Metropolis-adjusted Langevin algorithm (denoted by MALA) are presented in Algorithm A2. Compared to the random-walk Metropolis–Hastings, MALA usually proposes moves into regions of higher probability, which are then more likely to be accepted.

We note that the step-size *h* for MALA is chosen such that the acceptance rate is approximately 0.5 following [45], while the step-size for LMC in the same setting should be smaller than the one for MALA [46].

### 4.2. Simulation Studies for Biliear Regression

We perform some numerical studies on simulated data to assess the performance of our proposed algorithms. All simulations were conducted using the R statistical software [47].

For fixed dimensions q=10,k=20 of the data, we vary n=100 and n=1000 to check the effect of the samples, whereas the dimensions of the coefficient matrix are varied by p=10 and p=100. The entries of the design matrices *X* and *Z* are independently simulated from the standard Gaussian N(0,1). Then, given a matrix $M^{*}$, we simulate the response matrix *Y* from model (1) whose entries of the noise matrix *E* are independent and identically sampled from N(0,1). We consider the following setups for the true coefficient matrix:

- Model I: The true coefficient matrix $M^{*}$ is a rank-2 matrix that is generated as $M^{*}=B_{1}B_{2}^{⊤}$ where $B_{1}\in R^{p\times 2},B_{2}\in R^{k\times 2}$ and all entries in $B_{1}$ and $B_{2}$ are independent and identically sampled from N(0,1).
- Model II: An approximate low-rank set up is studied. This series of simulations is similar to the Model I, except that the true coefficient is no longer rank 2, but it can be well approximated by a rank 2 matrix:
  $$M^{*}=2\cdot B_{1}B_{2}^{⊤}+U,$$
  where *U* is a matrix whose entries are independent and identically sampled from N(0,0.1).

We compare our approaches denoted by LMC and MALA against the (generalized) ordinary least square [2], denoted by OLS. The OLS is defined as follows:

$${\hat{M}}_{\mathrm{OLS}}={\left(X^{⊤}X\right)}^{†}X^{⊤}YZ^{⊤}{\left(ZZ^{⊤}\right)}^{†}$$

where $A^{†}$ denotes the Moore–Penrose inverse of matrix *A*. We fixed λ=nq,τ=1, and the LMC and MALA methods are initiated at the OLS estimator and are run with 10,000 iterations, where the first 1000 steps are removed as burn-in periods.

The evaluations are performed by using the mean squared estimation error (Est) and the normalized (relative) mean square error (Nmse):

$$\mathrm{Est}:=∥\hat{M}-M^{*}{∥}_{F}^{2}/\left(pk\right),\;\mathrm{Nmse}:=∥\hat{M}-M^{*}{∥}_{F}^{2}/{∥M^{*}∥}_{F}^{2},$$

and the prediction error (Pred) as

$$\mathrm{Pred}:=∥X\left(\hat{M}-M^{*}\right){Z∥}_{F}^{2}/\left(nq\right),$$

where $\hat{M}$ here is one of the estimators for LMC, MALA or OLS. We report the averages and the standard deviation of these errors over 100 data replications.

The results of our study are presented in Table 1 and Table 2. From the tables, it can be observed that our proposed methods perform similarly to the OLS method. However, the estimation method obtained from the MALA algorithm often results in smaller prediction errors, particularly in high-dimensional settings. This advantage is even more pronounced when the method is applied in the context of inductive matrix completion, as discussed in the next subsection.

### 4.3. Simulation Studies for Inductive Matrix Completion

The simulation settings for inductive matrix completion are similar to the settings for bilinear regression, Section 4.2. However, after obtaining the response matrix *Y*, we remove uniformly at random κ=10% and κ=30% of the entries of *Y*. Here, κ denotes the missing rate. We denote the response matrix with missing entries by $Y_{\mathrm{miss}}$.

As in the context of inductive matrix completion, we only observe the response matrix with missing entries, $Y_{\mathrm{miss}}$, and thus we cannot construct the OLS estimator as in the case of bilinear regression. For this purpose, we first impute the missing entries in $Y_{\mathrm{miss}}$ by using the R package softImpute [48], where the rank of $M^{*}$ is specified as the true rank for matrix $Y_{\mathrm{miss}}$. We denote the resulting imputed matrix by $Y_{\mathrm{imp}}$.

We compare our approaches denoted by LMC and MALA against the (imputed and generalized) ordinary least square, denoted by OLS_imp. The OLS_imp is defined as follows:

$${\hat{M}}_{\mathrm{OLS}\_\mathrm{imp}}={\left(X^{⊤}X\right)}^{†}X^{⊤}Y_{\mathrm{imp}}Z^{⊤}{\left(ZZ^{⊤}\right)}^{†}$$

where $A^{†}$ denotes the Moore–Penrose inverse of matrix *A*. The LMC and MALA methods are initiated at the OLS_imp estimator and are run with 10,000 iterations, where the first 1000 steps are removed as burn-in periods.

As previously discussed in Section 4.2, we present the averages and the standard deviation of the mean squared estimation error (Est), the normalized (relative) mean square error (Nmse), and the prediction error (Pred) over 100 data replications in our results.

The results are detailed in Table 3 and Table 4. It is evident from these tables that the results obtained from our MALA method surpass those of the other methods in terms of prediction error in most of the settings considered. This advantage becomes more pronounced as the missing rate in the response matrix increases. Additionally, it is worth noting that our MALA method is robust and performs well in the approximate low-rank setting (model II), while the OLS and LMC methods do not.

## 5. Discussion and Conclusions

In this paper, we focus on the problem of bilinear regression and its extension, the problem of inductive matrix completion, where the response matrix contains missing data. We propose a novel approach that combines elements of Bayesian statistics with a quasi-likelihood method. Our proposed method first addresses the problem of bilinear regression using a quasi-Bayesian approach and then adapts this approach to the problem of inductive matrix completion. By making use of a low-rankness assumption and leveraging the powerful PAC-Bayes bound technique, we provide statistical properties for our proposed estimators and for the quasi-posteriors.

Our proposed method includes an efficient gradient-based sampling algorithm that is designed to sample from the (quasi) posterior distribution. This algorithm allows for the approximate computation of mean estimators. These methods, referred to as LMC and MALA, were tested in various simulation studies and were found to perform well when compared to the ordinary least squared method. The ability to accurately sample from the (quasi) posterior distribution and compute mean estimators makes these methods a valuable tool for data analysis and modeling.

There are still some unresolved issues that require further investigation. One of these is the presence of missing data in the covariate matrices *X* and *Z*. This can have a significant impact on the analysis and may lead to biased results. Another area that needs further exploration is the assumption of independence and identically distributed data. In some cases, this assumption may not hold, and alternative models that allow for a dispersion matrix may be needed. These are potential topics for future research to address and further our understanding of these issues.

## Figures and Tables

**Table 1:** Simulation results on simulated data in Model I in bilinear regression, with fixed *q* = 10, *k* = 20, for different methods, with their standard error in parentheses over 100 replications. (Est: average of estimation errors; Pred: average of prediction errors; Nmse: average of normalized estimation errors).

		Errors	LMC	MALA	OLS
n = 100		Est	1.0053 (0.5480)	1.0342 (0.5559)	1.0052 (0.5478)
	p=10	Pred	0.1138 (0.0171)	0.0985 (0.0151)	0.1014 (0.0154)
		Nmse	0.4931 (0.1178)	0.5100 (0.1207)	0.4930 (0.1178)
		Est	1.3544 (0.5867)	1.3384 (0.5836)	1.3544 (0.5867)
	p=100	Pred	1.0066 (0.0430)	0.8761 (0.0756)	1.0030 (0.0424)
		Nmse	0.7049 (0.2944)	0.6963 (0.2927)	0.7049 (0.2944)
n = 1000		Est	1.0776 (0.5671)	1.0900 (0.5670)	1.0776 (0.5671)
	p=10	Pred	0.0099 (0.0013)	0.0099 (0.0013)	0.0099 (0.0013)
		Nmse	0.5185 (0.1198)	0.5264 (0.1219)	0.5185 (0.1198)
		Est	0.9662 (0.3240)	0.9688 (0.3244)	0.9662 (0.3240)
	p=100	Pred	0.0999 (0.0051)	0.0989 (0.0049)	0.0998 (0.0051)
		Nmse	0.4961 (0.1183)	0.4976 (0.1191)	0.4961 (0.1183)

**Table 2:** Simulation results on simulated data in Model II (approximate low-rank) in bilinear regression, with fixed q=10,k=20, for different methods, with their standard error in parentheses over 100 replications. (Est: average of estimation errors; Pred: average of prediction errors; Nmse: average of normalized estimation errors).

		Errors	LMC	MALA	OLS
n = 100		Est	4.0731 (1.828)	4.0989 (1.821)	4.0731 (1.828)
	p=10	Pred	0.1090 (0.0160)	0.0969 (0.0140)	0.0987 (0.0145)
		Nmse	0.5119 (0.1226)	0.5162 (0.1241)	0.5118 (0.1226)
		Est	4.6047 (1.812)	4.6038 (1.813)	4.6047 (1.812)
	p=100	Pred	1.0062 (0.0462)	1.0597 (0.0495)	1.0006 (0.0469)
		Nmse	0.5801 (0.1942)	0.5800 (0.1941)	0.5801 (0.1942)
n = 1000		Est	3.6733 (1.606)	3.6884 (1.606)	3.6733 (1.606)
	p=10	Pred	0.0098 (0.0015)	0.0098 (0.0015)	0.0098 (0.0015)
		Nmse	0.4812 (0.1271)	0.4835 (0.1260)	0.4813 (0.1271)
		Est	3.9972 (1.375)	3.9986 (1.376)	3.9972 (1.375)
	p=100	Pred	0.1000 (0.0043)	0.1032 (0.0057)	0.0999 (0.0043)
		Nmse	0.5013 (0.1061)	0.5014 (0.1063)	0.5013 (0.1062)

**Table 3:** Simulation results on simulated data in Model I in inductive matrix completion, with fixed q=10,k=20, for different methods, with their standard error in parentheses over 100 replications. (κ is the missing rate; Est: average of estimation errors; Pred: average of prediction errors; Nmse: average of normalized estimation errors).

		Errors	LMC	MALA	OLS_imp
n = 100 κ = 10%		Est	1.0559 (0.5060)	1.0803 (0.5122)	1.0559 (0.5060)
	p=10	Pred	0.1028 (0.0193)	0.1082 (0.0143)	0.1020 (0.0197)
		Nmse	0.4986 (0.1116)	0.5139 (0.1197)	0.4986 (0.1116)
		Est	1.4008 (0.8555)	1.3987 (0.8542)	1.4009 (0.8555)
	p=100	Pred	1.2250 (0.4568)	1.4468 (0.4137)	1.2252 (0.4570)
		Nmse	0.7148 (0.3591)	0.7136 (0.3581)	0.7148 (0.3591)
n = 100 κ = 30%		Est	1.0432 (0.4963)	1.0917 (0.5085)	1.0432 (0.4963)
	p=10	Pred	0.2402 (0.2705)	0.1447 (0.0204)	0.2446 (0.2780)
		Nmse	0.5242 (0.1257)	0.5538 (0.1335)	0.5242 (0.1257)
		Est	1.6242 (0.8179)	1.6224 (0.8169)	1.6242 (0.8179)
	p=100	Pred	9.8879 (14.11)	10.807 (13.84)	9.8901 (14.11)
		Nmse	0.7993 (0.3340)	0.7985 (0.3334)	0.7993 (0.3340)
n = 1000 κ = 10%		Est	0.9810 (0.4532)	0.9882 (0.4478)	0.9810 (0.4532)
	p=10	Pred	0.0114 (0.0033)	0.0112 (0.0015)	0.0114 (0.0033)
		Nmse	0.4933 (0.1076)	0.4984 (0.1075)	0.4933 (0.1076)
		Est	1.0063 (0.3465)	1.0088 (0.3471)	1.0063 (0.3465)
	p=100	Pred	0.1902 (0.1758)	0.1116 (0.0049)	0.1902 (0.1759)
		Nmse	0.5069 (0.1049)	0.5082 (0.1050)	0.5069 (0.1049)
n = 1000 κ = 30%		Est	1.0110 (0.4886)	1.0223 (0.4872)	1.0110 (0.4886)
	p=10	Pred	0.0539 (0.0599)	0.0141 (0.0019)	0.0540 (0.0599)
		Nmse	0.5129 (0.1030)	0.5206 (0.1043)	0.5129 (0.1030)
		Est	1.0291 (0.3567)	1.0312 (0.3555)	1.0291 (0.3567)
	p=100	Pred	1.7529 (1.914)	0.1475 (0.0078)	1.7530 (1.913)
		Nmse	0.5054 (0.1055)	0.5067 (0.1053)	0.5054 (0.1055)

**Table 4:** Simulation results on simulated data in Model II (approximate low-rank) in inductive matrix completion, with fixed q=10,k=20, for different methods, with their standard error in parentheses over 100 replications. (κ is the missing rate; Est: average of estimation errors; Pred: average of prediction errors; Nmse: average of normalized estimation errors).

		Errors	LMC	MALA	OLS_imp
n = 100 imis 10%		Est	3.8319 (1.691)	3.8749 (1.719)	3.8319 (1.690)
	p=10	Pred	0.1604 (0.1271)	0.1092 (0.0153)	0.1598 (0.1322)
		Nmse	0.5116 (0.1154)	0.5169 (0.1147)	0.5116 (0.1155)
		Est	5.9500 (2.834)	5.9452 (2.835)	5.9500 (2.834)
	p=100	Pred	4.7640 (5.272)	4.6964 (5.515)	4.7658 (5.275)
		Nmse	0.7313 (0.3454)	0.7307 (0.3455)	0.7313 (0.3454)
n = 100 imis 30%		Est	4.1838 (1.850)	4.2535 (1.859)	4.1839 (1.850)
	p=10	Pred	0.7221 (0.7562)	0.1498 (0.0183)	0.7371 (0.7741)
		Nmse	0.5182 (0.1128)	0.5283 (0.1147)	0.5182 (0.1128)
		Est	7.1589 (4.084)	7.1558 (4.083)	7.1589 (4.084)
	p=100	Pred	39.899 (52.40)	40.233 (51.76)	39.908 (52.41)
		Nmse	0.8998 (0.3821)	0.8994 (0.3820)	0.8998 (0.3821)
n = 1000 imis 10%		Est	3.9618 (1.678)	3.9788 (1.677)	3.9618 (1.678)
	p=10	Pred	0.0409 (0.0269)	0.0110 (0.0015)	0.0409 (0.0269)
		Nmse	0.4968 (0.1196)	0.4989 (0.1195)	0.4968 (0.1196)
		Est	4.1153 (1.295)	4.1163 (1.294)	4.1153 (1.295)
	p=100	Pred	1.0250 (0.9988)	0.1135 (0.0051)	1.0250 (0.9988)
		Nmse	0.5060 (0.1096)	0.5062 (0.1096)	0.5060 (0.1096)
n = 1000 imis 30%		Est	4.1647 (1.990)	4.1836 (1.995)	4.1647 (1.990)
	p=10	Pred	0.4615 (0.3497)	0.0141 (0.0017)	0.4616 (0.3498)
		Nmse	0.4905 (0.1157)	0.4933 (0.1171)	0.4905 (0.1157)
		Est	4.0578 (1.400)	4.0565 (1.397)	4.0578 (1.400)
	p=100	Pred	8.5608 (6.419)	0.1538 (0.0069)	8.5609 (6.419)
		Nmse	0.4944 (0.1184)	0.4943 (0.1180)	0.4944 (0.1184)

## Data Availability

The R codes used in the numerical experiments are available at: https://github.com/tienmt/blr_imc (accessed on 10 February 2023).

## Appendix A. Appendix: Proofs

The main technique for our proofs is the oracle-type PAC-Bayes bounds, in the spirit of [35]. We start with a few preliminary lemmas.

### Appendix A.1. Preliminary Lemmas

First, we state a version of Bernstein’s inequality from Proposition 2.9 page 24 in [49].

**Lemma** **A1** (Bernstein’s inequality). *Let $U_{1}$, …, $U_{n}$ be independent real valued random variables. Let us assume that there are two constants v and w such that $\sum_{i=1}^{n}E\left[U_{i}^{2}\right]\leq v$ and for all integers k≥3, $\sum_{i=1}^{n}E\left[{\left(U_{i}\right)}^{k}\right]\leq v\frac{k!w^{k-2}}{2}.$ Then, for any ζ∈(0,1/w),*

$$E\exp\left[\zeta \sum_{i=1}^{n}\left[U_{i}-E\left(U_{i}\right)\right]\right]\leq \exp\left(\frac{v\zeta^{2}}{2(1-w\zeta )}\right).$$

Another basic tool to derive the PAC-Bayes bounds is Donsker and Varadhan’s variational inequality, see Lemma 1.1.3 in Catoni [17] for a proof (among others). From now, for any $\Theta \subset R^{n_{1}\times n_{2}}$, we let P(Θ) denote the set of all probability distributions on Θ equipped with the Borel σ-algebra. For $\left(\mu ,\nu \right)\in P{\left(\Theta \right)}^{2}$, the Kullback–Leibler divergence is defined by $K\left(\nu ,\mu \right)=\int \log\left(\frac{d\nu }{d\mu }\left(\theta \right)\right)\nu \left(d\theta \right)$ if ν admits a density $\frac{d\nu }{d\mu }$ with respect to μ, and K(ν,μ)=+∞ otherwise.

**Lemma** **A2** (Donsker and Varadhan’s variational formula). *Let μ∈P(Θ). For any measurable, bounded function h:Θ→R, we have*

$$\log\int e^{h(\theta )}\mu \left(d\theta \right)=\underset{\rho \in P(\Theta )}{\sup}\left[\int h(\theta )\rho (d\theta )-K(\rho ,\mu )\right].$$

*Moreover, the supremum with respect to ρ in the right-hand side is reached for the Gibbs measure $\mu_{h}$ defined by its density with respect to μ*

(A1)
$$d\mu_{h}\left(\theta \right)=\frac{e^{h(\theta )}d\mu }{\int e^{h(\vartheta )}\mu \left(d\vartheta \right)}.$$

These two lemmas are the only tools we need to prove Theorems 1 and 2. Their proofs are quite similar, with a few differences. For the sake of simplicity, we will state the common parts of the proofs as a separate result in Lemma A3. Note that the proof of this lemma will use Lemmas A1 and A2.

**Lemma** **A3.**
*Under Assumptions 1 and 2, put*

(A2)
$$\alpha =\left(\lambda -\frac{\lambda^{2}C_{1}}{2nq(1-\frac{C_{2}\lambda }{nq})}\right)\;\;\mathrm{and}\;\;\beta =\left(\lambda +\frac{\lambda^{2}C_{1}}{2nq(1-\frac{C_{2}\lambda }{nq})}\right).$$

*Then, for any ε∈(0,1), and $\lambda \in (0,nq/C_{2})$,*

(A3)
$$\begin{array}{c} E[\int \exp\{\alpha \left(R\left(M\right)-R\left(M^{*}\right)\right)+\lambda \left(-r\left(M\right)+r\left(M^{*}\right)\right)-\log\left[\frac{d{\hat{\rho }}_{\lambda }}{d\pi }\left(M\right)\right]-\\ \log\frac{2}{\varepsilon }\}{\hat{\rho }}_{\lambda }\left(dM\right)]\leq \frac{\varepsilon }{2} \end{array}$$

*and*

(A4)
$$\begin{array}{c} E\underset{\rho \in P(R^{p\times k})}{\sup}\exp\left[\beta \left(-\int Rd\rho +R(M^{*})\right)+\lambda \left(\int rd\rho -r(M^{*})\right)-K\left(\rho ,\pi \right)-\log\frac{2}{\varepsilon }\right]\leq \frac{\varepsilon }{2}. \end{array}$$

**Proof** **of** **Lemma** **A3.** We prove the first inequality (A10) as follows. Fix any *M* with ${∥XMZ∥}_{\infty }\leq C$ and put

$$T_{ij}={\left(Y_{ij}-{\left(XM^{*}Z\right)}_{ij}\right)}^{2}-{\left(Y_{ij}-{\left(\Pi_{C}\left(XMZ\right)\right)}_{ij}\right)}^{2}.$$

Note that the random variables $T_{ij}$ with i=1,⋯,n;j=1,⋯,q are independent by construction. We have

$$\begin{array}{cc} \sum_{i=1}^{n}\sum_{j=1}^{q}E\left[T_{ij}^{2}\right]\; & =\sum_{i=1}^{n}\sum_{j=1}^{q}E\left[{\left(2Y_{ij}-{\left(XM^{*}Z\right)}_{ij}-\Pi_{C}{\left(XMZ\right)}_{ij}\right)}^{2}{\left({\left(XM^{*}Z\right)}_{ij}-\Pi_{C}{\left(XMZ\right)}_{ij}\right)}^{2}\right]\\ \; & =\sum_{i=1}^{n}\sum_{j=1}^{q}E\left[{\left(2E_{ij}+{\left(XM^{*}Z\right)}_{ij}-\Pi_{C}{\left(XMZ\right)}_{ij}\right)}^{2}{\left({\left(XM^{*}Z\right)}_{ij}-\Pi_{C}{\left(XMZ\right)}_{ij}\right)}^{2}\right]\\ \; & \leq \sum_{i=1}^{n}\sum_{j=1}^{q}E\left[8\left[E_{ij}^{2}+C^{2}\right]{\left[{\left(XM^{*}Z\right)}_{ij}-\Pi_{C}{\left(XMZ\right)}_{ij}\right]}^{2}\right]\\ \; & \leq \sum_{i=1}^{n}\sum_{j=1}^{q}8\left[\sigma^{2}+C^{2}\right]E{\left[{\left(XM^{*}Z\right)}_{ij}-{\left(XMZ\right)}_{ij}\right]}^{2}\\ \; & \leq 8nq\left(\sigma^{2}+C^{2}\right)\left[R\left(M\right)-R\left(M^{*}\right)\right]\\ \; & =nqC_{1}\left[R\left(M\right)-R\left(M^{*}\right)\right]=:v\left(M,M^{*}\right). \end{array}$$

Next we have, for any integer k≥3, that

$$\begin{array}{cc} \; & \sum_{i=1}^{n}\sum_{j=1}^{q}E\left[{\left(T_{ij}\right)}^{k}\right]\\ \leq & \sum_{i=1}^{n}\sum_{j=1}^{q}E\left[{\left|2Y_{ij}-{\left(XM^{*}Z\right)}_{ij}-\Pi_{C}{\left(XMZ\right)}_{ij}\right|}^{k}{\left|{\left(XM^{*}Z\right)}_{ij}-\Pi_{C}{\left(XMZ\right)}_{ij}\right|}^{k}\right]\\ \leq & \sum_{i=1}^{n}\sum_{j=1}^{q}E\left[2^{k-1}\left[|2E_{ij}{|}^{k}+{\left(2C\right)}^{k}\right]{\left|{\left(XM^{*}Z\right)}_{ij}-\Pi_{C}{\left(XMZ\right)}_{ij}\right|}^{k}\right]\\ \leq & \sum_{i=1}^{n}\sum_{j=1}^{q}E\left[2^{2k-1}\left(|E_{ij}{|}^{k}+C^{k}\right){\left(2C\right)}^{k-2}{\left|{\left(XM^{*}Z\right)}_{ij}-\Pi_{C}{\left(XMZ\right)}_{ij}\right|}^{2}\right]\\ \leq & 2^{2k-1}\left[\sigma^{2}k!\xi^{k-2}+C^{k}\right]{\left(2C\right)}^{k-2}\sum_{i=1}^{n}\sum_{j=1}^{q}E{\left|{\left(XM^{*}Z\right)}_{ij}-{\left(XMZ\right)}_{ij}\right|}^{2}\\ \leq & \frac{2^{3k-3}\left[\sigma^{2}k!\xi^{k-2}+C^{k}\right]C^{k-2}}{8(\sigma^{2}+C^{2})}v\left(M,M^{*}\right)\\ \leq & \frac{2^{3k-6}\left[\sigma^{2}\xi^{k-2}+C^{k}\right]C^{k-2}}{\left(\sigma^{2}+C^{2}\right)}k!v\left(M,M^{*}\right)\\ \leq & 2^{3k-5}\left[\xi^{k-2}+C^{k-2}\right]C^{k-2}k!v\left(M,M^{*}\right)\\ \leq & 2^{3k-4}\max{\left(\xi ,C\right)}^{k-2}C^{k-2}k!v\left(M,M^{*}\right)\\ = & {\left[2^{3}\max\left(\xi ,C\right)C\right]}^{k-2}2^{2}k!v\left(M,M^{*}\right) \end{array}$$

and use the fact that, for any k≥3, $2^{2}\leq 2^{3(k-2)}/2$ to obtain

$$\begin{array}{c} \sum_{i=1}^{n}\sum_{j=1}^{q}E\left[{\left(T_{ij}\right)}^{k}\right]\leq \frac{{\left[2^{6}\max\left(\xi ,C\right)C\right]}^{k-2}k!v\left(M,M^{*}\right)}{2}=v\left(M,M^{*}\right)\frac{k!C_{2}^{k-2}}{2}. \end{array}$$

Thus, we can apply Lemma A1 with $U_{i}:=T_{i}$, $v:=v(M,M^{*})$, $w:=C_{2}$ and ζ:=λ/nq. We obtain, for any $\lambda \in \left(0,nq/w\right)=\left(0,nq/C_{2}\right)$,

$$\begin{array}{cc} E\exp\left[\lambda \left(R\left(M\right)-R\left(M^{*}\right)-r\left(M\right)+r\left(M^{*}\right)\right)\right]\; & \leq \exp\left[\frac{v\lambda^{2}}{2{\left(nq\right)}^{2}\left(1-\frac{w\lambda }{nq}\right)}\right]\\ \; & =\exp\left[\frac{C_{1}\left[R\left(M\right)-R\left(M^{*}\right)\right]\lambda^{2}}{2nq(1-\frac{C_{2}\lambda }{nq})}\right]. \end{array}$$

Rearranging terms, and using the definition of α in (A2),

$$E\exp\left[\alpha \left(R\left(M\right)-R\left(M^{*}\right)\right)+\lambda \left(-r\left(M\right)+r\left(M^{*}\right)\right)\right]\leq 1.$$

Multiplying both sides by ε/2 and then integrating with respect to the probability distribution π(.), we obtain

$$\int E\left[\exp\left\{\alpha \left(R\left(M\right)-R\left(M^{*}\right)\right)+\lambda \left(-r\left(M\right)+r\left(M^{*}\right)\right)-\log\frac{2}{\varepsilon }\right\}\right]\pi \left(dM\right)\leq \frac{\varepsilon }{2}.$$

Next, Fubini’s theorem gives

$$E\left[\int \exp\left[\alpha \left(R\left(M\right)-R\left(M^{*}\right)\right)+\lambda \left(-r\left(M\right)+r\left(M^{*}\right)\right)-\log\frac{2}{\varepsilon }\right]\pi \left(dM\right)\right]\leq \frac{\varepsilon }{2}.$$

and note that for any measurable function *h*,

$$\begin{array}{c} \int \exp\left[h\left(M\right)\right]\pi \left(dM\right)=\int \exp\left[h\left(M\right)-\log\frac{d{\hat{\rho }}_{\lambda }}{d\pi }\left(M\right)\right]{\hat{\rho }}_{\lambda }\left(dM\right) \end{array}$$

to obtain (A3). Let us now prove (A4). Here again, we start with an application of Lemma A1, but this time with $U_{i}:=-T_{i}$ (we keep $v:=v(M,M^{*})$, $w:=C_{2}$ and ζ:=λ/nq). We obtain, for any $\lambda \in (0,nq/C_{2})$,

$$E\exp\left[\lambda \left(r\left(M\right)+r\left(M^{*}\right)-R\left(M\right)+R\left(M^{*}\right)\right)\right]\leq \exp\left[\frac{C_{1}\left[R\left(M\right)-R\left(M^{*}\right)\right]\lambda^{2}}{2nq(1-\frac{C_{2}\lambda }{nq})}\right].$$

Rearranging terms, using the definition of β in (A2) and multiplying both sides by ε/2, we obtain

$$E\exp\left[\beta \left(-R\left(M\right)+R\left(M^{*}\right)\right)+\lambda \left(r\left(M\right)-r\left(M^{*}\right)\right)-\log\frac{2}{\varepsilon }\right]\leq \frac{\varepsilon }{2}.$$

We integrate with respect to π and use Fubini to obtain

$$E\left[\int \exp\left[\beta \left(-R\left(M\right)+R\left(M^{*}\right)\right)+\lambda \left(r\left(M\right)-r\left(M^{*}\right)\right)-\log\frac{2}{\varepsilon }\right]\pi \left(dM\right)\right]\leq \frac{\varepsilon }{2}.$$

Here, we use a different argument from the proof of the first inequality: we use Lemma A2 on the integral, and this gives directly (A4).    □

Finally, in both proofs, we will use quite often distributions $\rho \in P(R^{p\times k})$ that will be defined as translations of the prior π. We introduce the following notation.

**Definition** **A1.**
*For any matrix $\bar{M}\in R^{p\times k}$, we define $\rho_{\bar{M}}\in P\left(R^{p\times k}\right)$ by*

$$\rho_{\bar{M}}\left(M\right)=\pi \left(\bar{M}-M\right).$$

The following technical lemmas from [31] will be useful in the proofs.

**Lemma** **A4** (Lemma 1 in [31]). *We have ${\int ∥M∥}_{F}^{2}\pi \left(dM\right)\leq pk\tau^{2}.$*

**Lemma** **A5** (Lemma 2 in [31]). *For any $\bar{M}\in R^{p\times k}$, we have*

$$K\left(\rho_{\bar{M}},\pi \right)\leq 2\mathrm{rank}\left(\bar{M}\right)\left(k+p+2\right)\log\left(1+\frac{∥\bar{M}{∥}_{F}}{\tau \sqrt{2\mathrm{rank}(\bar{M})}}\right)$$

*with the convention 0log(1+0/0)=0.*

### Appendix A.2. Proof of Theorem 1

**Proof** **of** **Theorem** **1.** An application of Jensen’s inequality on inequality (A3) yields

$$E\exp\left[\alpha \left(\int Rd{\hat{\rho }}_{\lambda }-R\left(M^{*}\right)\right)+\lambda \left(-\int rd{\hat{\rho }}_{\lambda }+r\left(M^{*}\right)\right)-K\left({\hat{\rho }}_{\lambda },\pi \right)-\log\frac{2}{\varepsilon }\right]\leq \frac{\varepsilon }{2}.$$

Using the standard Chernoff’s trick to transform an exponential moment inequality into a deviation inequality, that is: $\exp\left(x\right)\geq 1_{R_{+}}\left(x\right)$, we obtain

(A5) $$\begin{array}{c} P\alpha \left(\int Rd{\hat{\rho }}_{\lambda }-R\left(M^{*}\right)\right)+\lambda \left(-\int rd{\hat{\rho }}_{\lambda }+r\left(M^{*}\right)\right)-K\left({\hat{\rho }}_{\lambda },\pi \right)-\log\frac{2}{\varepsilon }]\geq 0\}\leq \frac{\varepsilon }{2} \end{array}$$

Using (2) we have

$$\begin{array}{cc} \int Rd{\hat{\rho }}_{\lambda }-R\left(M^{*}\right)\; & =\frac{1}{nq}\int {∥\Pi_{C}\left(XMZ\right)-XM^{*}Z∥}_{F}^{2}{\hat{\rho }}_{\lambda }\left(dM\right)\\ \; & \geq \frac{1}{nq}{∥\int \Pi_{C}\left(XMZ\right){\hat{\rho }}_{\lambda }\left(dM\right)-XM^{*}Z∥}_{F}^{2}\\ \; & \geq \frac{1}{nq}{∥{\hat{XMZ}}_{\lambda }-XM^{*}Z∥}_{F}^{2} \end{array}$$

where we used Jensen’s inequality in the second line, and the definition of ${\hat{XMZ}}_{\lambda }$ from the second to the third line. Plugging this into our probability bound (A5), and dividing both sides by α, we obtain

$$P\left\{\frac{1}{nq}{∥{\hat{XMZ}}_{\lambda }-XM^{*}Z∥}_{F}^{2}\leq \frac{\int rd{\hat{\rho }}_{\lambda }-r\left(M^{*}\right)+\frac{1}{\lambda }\left[K\left({\hat{\rho }}_{\lambda },\pi \right)+\log\frac{2}{\varepsilon }\right]}{\frac{\alpha }{\lambda }}\right\}\geq 1-\frac{\varepsilon }{2}$$

under the additional condition that λ is such that α>0, which we will assume from now (note that this is satisfied by $\lambda^{*}$). Using Lemma A2, we can rewrite this as

(A6) $$P\left\{\frac{1}{nq}{∥{\hat{XMZ}}_{\lambda }-XM^{*}Z∥}_{F}^{2}\leq \underset{\rho \in P(R^{p\times k})}{\inf}\frac{\int rd\rho -r\left(M^{*}\right)+\frac{1}{\lambda }\left[K\left(\rho ,\pi \right)+\log\frac{2}{\varepsilon }\right]}{\frac{\alpha }{\lambda }}\right\}\geq 1-\frac{\varepsilon }{2}.$$

We consider now the consequences of the second inequality in Lemma A3, that is (A4). With Chernoff’s trick and rearranging terms a little, we obtain

$$\begin{array}{c} P\left\{\forall \rho \in P\left(R^{p\times k}\right),\int rd\rho -r\left(M^{*}\right)\leq \frac{\beta }{\lambda }\left[\int Rd\rho -R(M^{*})\right]+\frac{1}{\lambda }\left[K\left(\rho ,\pi \right)+\log\frac{2}{\varepsilon }\right]\right\}\geq 1-\frac{\varepsilon }{2}. \end{array}$$

which we can rewrite as, $\forall \rho \in P(R^{p\times k}),$ with probability at least $1-\frac{\varepsilon }{2}$,

(A7) $$\begin{array}{c} \int rd\rho -r\left(M^{*}\right)\leq \frac{\beta }{\lambda }\int \frac{1}{nq}{∥\Pi_{C}\left(XMZ\right)-XM^{*}Z∥}_{F}^{2}\rho \left(dM\right)+\frac{1}{\lambda }\left[K\left(\rho ,\pi \right)+\log\frac{2}{\varepsilon }\right]. \end{array}$$

Combining (A7) and (A6) with a union bound argument gives the following bound, with probability of at least 1−ε,

$$\begin{array}{c} \frac{1}{nq}{∥{\hat{XMZ}}_{\lambda }-XM^{*}Z∥}_{F}^{2}\\ \leq \underset{\rho \in P(R^{p\times k})}{\inf}\frac{\beta \int \frac{1}{nq}{∥\Pi_{C}\left(XMZ\right)-XM^{*}Z∥}_{F}^{2}\rho \left(dM\right)+2\left[K\left(\rho ,\pi \right)+\log\frac{2}{\varepsilon }\right]}{\alpha }. \end{array}$$

Noting that, for any (i,j), ${\left(XM^{*}Z\right)}_{i,j}\in \left[-C,C\right]$ implies that

$$|{\left(\Pi_{C}\left(XMZ\right)\right)}_{i,j}-{\left(XM^{*}Z\right)}_{i,j}\left|\leq \right|{\left(XMZ\right)}_{i,j}-{\left(XM^{*}Z\right)}_{i,j}|$$

and thus

$$\begin{array}{c} \frac{1}{nq}{∥{\hat{XMZ}}_{\lambda }-XM^{*}Z∥}_{F}^{2}\leq \underset{\rho \in P(R^{p\times k})}{\inf}\frac{\beta \int \frac{1}{nq}{∥XMZ-XM^{*}Z∥}_{F}^{2}\rho \left(dM\right)+2\left[K\left(\rho ,\pi \right)+\log\frac{2}{\varepsilon }\right]}{\alpha }. \end{array}$$

The end of the proof consists in making the right-hand side in the inequality more explicit. In order to do so, we restrict the infimum bound above to the distributions given by Definition A1:

(A8) $$\begin{array}{c} P\{\frac{1}{nq}{∥{\hat{XMZ}}_{\lambda }-XM^{*}Z∥}_{F}^{2}\\ \leq \underset{\bar{M}\in R^{p\times k}}{\inf}\frac{\beta \int \frac{1}{nq}{∥XMZ-XM^{*}Z∥}_{F}^{2}\rho_{\bar{M}}\left(dM\right)+2\left[K\left(\rho_{\bar{M}},\pi \right)+\log\frac{2}{\varepsilon }\right]}{\alpha }\}\geq 1-\varepsilon . \end{array}$$

We see immediately that Dalalyan’s lemma will be extremely useful for that. First, Lemma A5 provides an upper bound on $K(\rho_{\bar{M}},\pi )$. Moreover,

$$\begin{array}{cc} \; & \int ∥XMZ-XM^{*}{Z∥}_{F}^{2}\rho_{\bar{M}}\left(dM\right)\\ \; & \leq \int ∥X\bar{M}Z-XM^{*}{Z-XMZ∥}_{F}^{2}\pi \left(dM\right)\\ \; & =∥X\bar{M}Z-XM^{*}{Z∥}_{F}^{2}-2\int \sum_{i,j}{\left(X\bar{M}Z-XM^{*}Z\right)}_{j,i}{\left(XMZ\right)}_{i,j}\pi \left(dM\right)+\int {∥XMZ∥}_{F}^{2}\pi \left(dM\right). \end{array}$$

The second term in the right-hand side is null because π is centered, and thus

$$\begin{array}{cc} \int ∥XMZ-XM^{*}{Z∥}_{F}^{2}\rho_{\bar{M}}\left(dM\right) & \leq ∥X\bar{M}Z-XM^{*}{Z∥}_{F}^{2}+\int {∥XMZ∥}_{F}^{2}\;\pi \left(dM\right)\\ & \leq ∥X\bar{M}Z-XM^{*}{Z∥}_{F}^{2}+{∥X∥}_{F}^{2}{∥Z∥}_{F}^{2}\int {∥M∥}_{F}^{2}\;\pi \left(dM\right)\\ & \leq ∥X\bar{M}Z-XM^{*}{Z∥}_{F}^{2}+{∥X∥}_{F}^{2}{∥Z∥}_{F}^{2}pk\tau^{2} \end{array}$$

where we used elementary properties of the Frobenius norm, and Lemma A4 in the last line. We can now plug this (and Lemma A5) back into (A8) to obtain

$$\begin{array}{c} P\{\frac{1}{nq}{∥{\hat{XMZ}}_{\lambda }-XM^{*}Z∥}_{F}^{2}\leq \underset{\bar{M}\in R^{p\times k}}{\inf}[\frac{\beta }{\alpha }\frac{1}{nq}∥X\bar{M}Z-XM^{*}{Z∥}_{F}^{2}+\frac{\beta }{\alpha }\frac{1}{nq}{∥X∥}_{F}^{2}{∥Z∥}_{F}^{2}pk\tau^{2}\\ +\frac{1}{\alpha }\left(4\mathrm{rank}\left(\bar{M}\right)\left(k+p+2\right)\log\left(1+\frac{∥\bar{M}{∥}_{F}}{\tau \sqrt{2\mathrm{rank}(\bar{M})}}\right)+2\log\frac{2}{\varepsilon }\right)]\}\geq 1-\varepsilon . \end{array}$$

We are now making the constants explicit. First, if $\lambda \leq nq/(2C_{2})$, then $2nq(1-C_{2}\lambda /nq)\geq np$ and thus

$$\frac{\beta }{\alpha }=\frac{1+\frac{\lambda C_{1}}{2nq(1-\frac{C_{2}\lambda }{nq})}}{1-\frac{\lambda C_{1}}{2nq(1-\frac{C_{2}\lambda }{nq})}}\leq \frac{1+\frac{\lambda C_{1}}{nq}}{1-\frac{\lambda C_{1}}{nq}}.$$

Then, $\lambda \leq \frac{nq\delta }{C_{1}\left(1+\delta \right)}$ leads to $\frac{\beta }{\alpha }\leq \left(1+\delta \right).$ Note that $\lambda^{*}=nq\min\left(1/\left(2C_{2}\right),\delta /\left[C_{1}\left(1+\delta \right)\right]\right)$ satisfies these two conditions, so from now, $\lambda =\lambda^{*}$. We also use the following:

$$\begin{array}{c} \frac{1}{\alpha }=\frac{1}{\lambda^{*}\left(1-\frac{\lambda^{*}C_{1}}{2nq(1-C_{2}\lambda^{*}/nq)}\right)}\leq \frac{\beta }{\lambda^{*}\alpha }\leq \frac{\left(1+\delta \right)}{nq\min(1/\left(2C_{2}\right),\delta /\left[C_{1}\left(1+\delta \right)\right])}\leq \frac{C_{1}{\left(1+\delta \right)}^{2}}{nq\delta }. \end{array}$$

So far the bound is:

$$\begin{array}{c} P\{\frac{1}{nq}{∥{\hat{XMZ}}_{\lambda^{*}}-XM^{*}Z∥}_{F}^{2}\leq \underset{\bar{M}\in R^{p\times k}}{\inf}[\frac{\left(1+\delta \right)}{nq}{∥X\bar{M}Z-XM^{*}Z∥}_{F}^{2}+\\ \frac{\left(1+\delta \right)}{nq}{∥X∥}_{F}^{2}{∥Z∥}_{F}^{2}pk\tau^{2}+\\ \frac{C_{1}{\left(1+\delta \right)}^{2}\left(4\mathrm{rank}\left(\bar{M}\right)\left(k+p+2\right)\log\left(1+\frac{∥\bar{M}{∥}_{F}}{\tau \sqrt{2\mathrm{rank}(\bar{M})}}\right)+2\log\frac{2}{\varepsilon }\right)}{nq\delta }]\}\geq 1-\varepsilon . \end{array}$$

In particular, with probability at least 1−ε, the choice $\tau^{2}=C_{1}\left(k+p\right)/{(nkq∥X∥}_{F}^{2}{∥Z∥}_{F}^{2})$ gives

$$\begin{array}{c} \frac{1}{nq}{∥{\hat{XMZ}}_{\lambda^{*}}-XM^{*}Z∥}_{F}^{2}\leq \underset{\bar{M}\in R^{p\times k}}{\inf}[\frac{\left(1+\delta \right)}{nq}{∥X\bar{M}Z-XM^{*}Z∥}_{F}^{2}+\frac{C_{1}\left(1+\delta \right)\left(k+p\right)}{nq}+\\ \frac{C_{1}{\left(1+\delta \right)}^{2}\left(4\mathrm{rank}\left(\bar{M}\right)\left(k+p+2\right)\log\left(1+\frac{{∥X∥}_{F}{∥Z∥}_{F}{∥\bar{M}∥}_{F}}{\sqrt{C_{1}}}\sqrt{\frac{nkq}{\left(k+p\right)\mathrm{rank}\left(\bar{M}\right)}}\right)+2\log\frac{2}{\varepsilon }\right)}{nq\delta }]. \end{array}$$

   □

### Appendix A.3. Proof of Theorem 2

**Proof** **of** **Theorem** **2.** We also start with an application of Lemma A3, and focus on (A3), applied to $\varepsilon :=\varepsilon_{n}$, that is:

$$\begin{array}{c} E[\int \exp\{\alpha \left(R\left(M\right)-R\left(M^{*}\right)\right)+\lambda \left(-r\left(M\right)+r\left(M^{*}\right)\right)-\log\left[\frac{d{\hat{\rho }}_{\lambda }}{d\pi }\left(M\right)\right]-\\ \log\frac{2}{\varepsilon_{n}}\}{\hat{\rho }}_{\lambda }\left(dM\right)]\leq \frac{\varepsilon_{n}}{2}. \end{array}$$

Using Chernoff’s trick, this gives

$$E\left[P_{M∼{\hat{\rho }}_{\lambda }}\left(M\in A_{n}\right)\right]\geq 1-\frac{\varepsilon_{n}}{2}$$

where

$$A_{n}=\left\{M:\alpha \left(R\left(M\right)-R\left(M^{*}\right)\right)+\lambda \left(-r\left(M\right)+r\left(M^{*}\right)\right)\leq \log\left[\frac{d{\hat{\rho }}_{\lambda }}{d\pi }\left(M\right)\right]+\log\frac{2}{\varepsilon_{n}}\right\}.$$

Using the definition of ${\hat{\rho }}_{\lambda }$, for $M\in A_{n}$ we have

$$\begin{array}{cc} \alpha \left(R\left(M\right)-R\left(M^{*}\right)\right) & \leq \lambda \left(r\left(M\right)-r\left(M^{*}\right)\right)+\log\left[\frac{d{\hat{\rho }}_{\lambda }}{d\pi }\left(M\right)\right]+\log\frac{2}{\varepsilon_{n}}\\ & \leq -\log\int \exp\left[-\lambda r(M)\right]\pi \left(dM\right)-\lambda r\left(M^{*}\right)+\log\frac{2}{\varepsilon_{n}}\\ & =\lambda \left(\int r\left(M\right){\hat{\rho }}_{\lambda }\left(dM\right)-r\left(M^{*}\right)\right)+K\left({\hat{\rho }}_{\lambda },\pi \right)+\log\frac{2}{\varepsilon_{n}}\\ & =\underset{\rho }{\inf}\left\{\lambda \left(\int r\left(M\right)\rho \left(dM\right)-r\left(M^{*}\right)\right)+K\left(\rho ,\pi \right)+\log\frac{2}{\varepsilon_{n}}\right\}. \end{array}$$

Now, let us define

$$B_{n}=\left\{\forall \rho :\beta \left(-\int Rd\rho +R(M^{*})\right)+\lambda \left(\int rd\rho -r(M^{*})\right)\leq K\left(\rho ,\pi \right)+\log\frac{2}{\varepsilon_{n}}\right\}.$$

Using (A4), we have that

$$E\left[1_{B_{n}}\right]\geq 1-\frac{\varepsilon_{n}}{2}.$$

We will now prove that, if λ is such that α>0,

$$E\left[P_{M∼{\hat{\rho }}_{\lambda }}\left(M\in M_{n}\right)\right]\geq E\left[P_{M∼{\hat{\rho }}_{\lambda }}\left(M\in A_{n}\right)1_{B_{n}}\right]$$

which, together with

$$\begin{array}{cc} E\left[P_{M∼{\hat{\rho }}_{\lambda }}\left(M\in A_{n}\right)1_{B_{n}}\right] & =E\left[\left(1-P_{M∼{\hat{\rho }}_{\lambda }}\left(M\notin A_{n}\right)\right)\left(1-1_{B_{n}^{c}}\right)\right]\\ & \geq E\left[1-P_{M∼{\hat{\rho }}_{\lambda }}\left(M\notin A_{n}\right)-1_{B_{n}^{c}}\right]\\ & \geq 1-\varepsilon_{n} \end{array}$$

will bring

$$E\left[P_{M∼{\hat{\rho }}_{\lambda }}\left(M\in M_{n}\right)\right]\geq 1-\varepsilon_{n}.$$

In order to do so, assume that we are on the set $B_{n}$, and let $M\in A_{n}$. Then,

$$\begin{array}{cc} \alpha \left(R\left(M\right)-R\left(M^{*}\right)\right) & \leq \underset{\rho }{\inf}\left\{\lambda \left(\int r\left(M\right)\rho \left(dM\right)-r\left(M^{*}\right)\right)+K\left(\rho ,\pi \right)+\log\frac{2}{\varepsilon_{n}}\right\}\\ & \leq \underset{\rho }{\inf}\left\{\beta \left(\int R\left(M\right)\rho \left(dM\right)-R\left(M^{*}\right)\right)+2K\left(\rho ,\pi \right)+2\log\frac{2}{\varepsilon_{n}}\right\} \end{array}$$

that is,

$$R\left(M\right)-R\left(M^{*}\right)\leq \underset{\rho \in P(R^{p\times k})}{\inf}\frac{\beta \left[\int Rd\rho -R(M^{*})\right]+2\left[K\left(\rho ,\pi \right)+\log\frac{2}{\varepsilon }\right]}{\alpha }$$

or, rewriting it in terms of norms,

$${∥\Pi_{C}\left(XMZ\right)-XM^{*}Z∥}_{F}^{2}\leq \underset{\bar{M}\in R^{p\times k}}{\inf}\frac{\beta \int ∥XMZ-XM^{*}{Z∥}_{F}^{2}\rho_{\bar{M}}\left(dM\right)+2\left[K\left(\rho_{\bar{M}},\pi \right)+\log\frac{2}{\varepsilon }\right]}{\alpha }.$$

We upper-bound the right-hand side exactly as in the proof of Theorem 1, which gives $M\in M_{n}$.   □

### Appendix A.4. Proof of Theorem 3

**Lemma** **A6.**
*Under Assumptions 1 and 3, put*

(A9)
$$\alpha^{\prime }=\left(\lambda -\frac{\lambda^{2}C_{1}^{\prime }}{2m(1-\frac{C_{2}^{\prime }\lambda }{m})}\right)\;\;\mathrm{and}\;\;\beta^{\prime }=\left(\lambda +\frac{\lambda^{2}C_{1}^{\prime }}{2m(1-\frac{C_{2}^{\prime }\lambda }{m})}\right).$$

*Then for any ε∈(0,1), and $\lambda \in (0,m/C_{2}^{\prime })$,*

(A10)
$$\begin{array}{c} E[\int \exp\{\alpha^{\prime }\left(R^{\prime }\left(M\right)-R^{\prime }\left(M^{*}\right)\right)+\lambda \left(-r^{\prime }\left(M\right)+r^{\prime }\left(M^{*}\right)\right)-\\ \log\left[\frac{d{\hat{\rho }}_{\lambda }^{\prime }}{d\pi }\left(M\right)\right]-\log\frac{2}{\varepsilon }\}{\hat{\rho }}_{\lambda }^{\prime }\left(dM\right)]\leq \frac{\varepsilon }{2} \end{array}$$

*and*

(A11)
$$\begin{array}{c} E\underset{\rho \in P(R^{p\times k})}{\sup}\exp\left[\beta^{\prime }\left(-\int R^{\prime }d\rho +R^{\prime }\left(M^{*}\right)\right)+\lambda \left(\int r^{\prime }d\rho -r^{\prime }\left(M^{*}\right)\right)-K\left(\rho ,\pi \right)-\log\frac{2}{\varepsilon }\right]\leq \frac{\varepsilon }{2}. \end{array}$$

**Proof** **of** **Lemma** **A6.** The inequality (A10) is proved in a similar way to the proof of Lemma A3. That is, we apply Lemma A1 to the following independent random variables

$$V_{i}={\left(Y_{i}-{\left(XM^{*}Z\right)}_{i}\right)}^{2}-{\left(Y_{i}-{\left(\Pi_{C}\left(XMZ\right)\right)}_{i}\right)}^{2},i=1,\ldots ,m.$$

The proof of the inequality (A11) is processed similar in the proof of Lemma A3 in which we apply Lemma A1 to the independent random variables $-V_{i},i=1,\ldots ,m.$    □

**Proof** **of** **Theorem** **3.** Similar to the proof of Theorem 1, until the (A6), and noting that using (6), we have

$$\begin{array}{cc} \int R^{\prime }d{\hat{\rho }}_{\lambda }-R^{\prime }\left(M^{*}\right) & =\int {∥\Pi_{C}\left(XMZ\right)-XM^{*}Z∥}_{F,\Pi }^{2}{\hat{\rho }}_{\lambda }^{\prime }\left(dM\right)\\ & \geq {∥\int \Pi_{C}\left(XMZ\right){\hat{\rho }}_{\lambda }^{\prime }\left(dM\right)-XM^{*}Z∥}_{F,\Pi }^{2}\\ & \geq {∥{\hat{XMZ}}_{\lambda }-XM^{*}Z∥}_{F,\Pi }^{2}, \end{array}$$

and thus, we obtain

(A12) $$P\left\{{∥{\hat{XMZ}}_{\lambda }-XM^{*}Z∥}_{F,\Pi }^{2}\leq \underset{\rho \in P(R^{p\times k})}{\inf}\frac{\int r^{\prime }d\rho -r^{\prime }\left(M^{*}\right)+\frac{1}{\lambda }\left[K\left(\rho ,\pi \right)+\log\frac{2}{\varepsilon }\right]}{\frac{\alpha^{\prime }}{\lambda }}\right\}\geq 1-\frac{\varepsilon }{2}.$$

We consider now the consequences of inequality (A11) in Lemma A6. With Chernoff’s trick and rearranging terms a little, we obtain $\forall \rho \in P(R^{p\times k})$, with probability at least $1-\frac{\varepsilon }{2}$,

(A13) $$\begin{array}{c} \int r^{\prime }d\rho -r^{\prime }\left(M^{*}\right)\leq \frac{\beta^{\prime }}{\lambda }\int {∥\Pi_{C}\left(XMZ\right)-XM^{*}Z∥}_{F,\Pi }^{2}\rho \left(dM\right)+\frac{1}{\lambda }\left[K\left(\rho ,\pi \right)+\log\frac{2}{\varepsilon }\right]. \end{array}$$

Combining (A13) and (A12) with a union bound argument gives the bound and noting that for any (i,j), ${\left(XM^{*}Z\right)}_{i,j}\in \left[-C,C\right]$ implies that $|{\left(\Pi_{C}\left(XMZ\right)\right)}_{i,j}-{\left(XM^{*}Z\right)}_{i,j}\left|\leq \right|{\left(XMZ\right)}_{i,j}-{\left(XM^{*}Z\right)}_{i,j}|$ and thus

$$\begin{array}{cc} P\{ & {∥{\hat{XMZ}}_{\lambda }-XM^{*}Z∥}_{F,\Pi }^{2}\\ \; & \leq \underset{\rho \in P(R^{p\times k})}{\inf}\frac{\beta^{\prime }\int {∥XMZ-XM^{*}Z∥}_{F,\Pi }^{2}\rho \left(dM\right)+2\left[K\left(\rho ,\pi \right)+\log\frac{2}{\varepsilon }\right]}{\alpha^{\prime }}\}\geq 1-\varepsilon . \end{array}$$

We are now making the right-hand side in the inequality more explicit. In order to do so, we restrict the infimum bound above to the distributions given by Definition A1:

(A14) $$\begin{array}{c} P\{{∥{\hat{XMZ}}_{\lambda }-XM^{*}Z∥}_{F,\Pi }^{2}\leq \\ \underset{\bar{M}\in R^{p\times k}}{\inf}\frac{\beta^{\prime }\int {∥XMZ-XM^{*}Z∥}_{F,\Pi }^{2}\rho_{\bar{M}}\left(dM\right)+2\left[K\left(\rho_{\bar{M}},\pi \right)+\log\frac{2}{\varepsilon }\right]}{\alpha^{\prime }}\}\geq 1-\varepsilon . \end{array}$$

We see immediately that Dalalyan’s lemma will be extremely useful for that. First, Lemma A5 provides an upper bound on $K(\rho_{\bar{M}},\pi )$. Moreover,

$$\begin{array}{cc} \; & \int ∥XMZ-XM^{*}{Z∥}_{F,\Pi }^{2}\rho_{\bar{M}}\left(dM\right)\\ \; & \leq \int ∥X\bar{M}Z-XM^{*}{Z-XMZ∥}_{F,\Pi }^{2}\pi \left(dM\right)\\ \; & =∥X\bar{M}Z-XM^{*}{Z∥}_{F,\Pi }^{2}-2\int \sum_{i,j}\Pi_{i,j}{\left(X\bar{M}Z-XM^{*}Z\right)}_{j,i}{\left(XMZ\right)}_{i,j}\pi \left(dM\right)+\\ \; & {\int ∥XMZ∥}_{F,\Pi }^{2}\pi \left(dM\right). \end{array}$$

The second term in the above right-hand side is null because π is centered, and thus

$$\begin{array}{cc} \; & \int ∥XMZ-XM^{*}{Z∥}_{F,\Pi }^{2}\rho_{\bar{M}}\left(dM\right)\\ \; & \leq ∥X\bar{M}Z-XM^{*}{Z∥}_{F,\Pi }^{2}+\int {∥XMZ∥}_{F,\Pi }^{2}\pi \left(dM\right)\\ \; & \leq ∥X\bar{M}Z-XM^{*}{Z∥}_{F,\Pi }^{2}+\int {∥XMZ∥}_{F}^{2}\;\pi \left(dM\right)\\ \; & \leq ∥X\bar{M}Z-XM^{*}{Z∥}_{F,\Pi }^{2}+{∥X∥}_{F}^{2}{∥Z∥}_{F}^{2}\int {∥M∥}_{F}^{2}\;\pi \left(dM\right)\\ \; & \leq ∥X\bar{M}Z-XM^{*}{Z∥}_{F,\Pi }^{2}+{∥X∥}_{F}^{2}{∥Z∥}_{F}^{2}pk\tau^{2} \end{array}$$

where we used the elementary properties of the Frobenius norm, and Lemma A4 in the last line. We can now plug this (and Lemma A5) back into (A14) to obtain:

$$\begin{array}{c} P\{{∥{\hat{XMZ}}_{\lambda }-XM^{*}Z∥}_{F,\Pi }^{2}\leq \underset{\bar{M}\in R^{p\times k}}{\inf}[\frac{\beta^{\prime }}{\alpha^{\prime }}∥X\bar{M}Z-XM^{*}{Z∥}_{F,\Pi }^{2}+\frac{\beta^{\prime }}{\alpha^{\prime }}{∥X∥}_{F}^{2}{∥Z∥}_{F}^{2}pk\tau^{2}\\ +\frac{1}{\alpha^{\prime }}\left(4\mathrm{rank}\left(\bar{M}\right)\left(k+p+2\right)\log\left(1+\frac{∥\bar{M}{∥}_{F}}{\tau \sqrt{2\mathrm{rank}(\bar{M})}}\right)+2\log\frac{2}{\varepsilon }\right)]\}\geq 1-\varepsilon . \end{array}$$

We are now making the constants explicit. First, if $\lambda \leq m/(2C_{2}^{\prime })$, then $2m(1-C_{2}^{\prime }\lambda /m)\geq m$ and thus

$$\frac{\beta^{\prime }}{\alpha^{\prime }}=\frac{1+\frac{\lambda C_{1}^{\prime }}{2m(1-\frac{C_{2}^{\prime }\lambda }{m})}}{1-\frac{\lambda C_{1}^{\prime }}{2m(1-\frac{C_{2}^{\prime }\lambda }{m})}}\leq \frac{1+\frac{\lambda C_{1}^{\prime }}{m}}{1-\frac{\lambda C_{1}^{\prime }}{m}}.$$

Then, $\lambda \leq \frac{m\delta }{C_{1}^{\prime }\left(1+\delta \right)}$ leads to $\frac{\beta^{\prime }}{\alpha^{\prime }}\leq \left(1+\delta \right).$ Note that $\lambda^{\prime *}=m\min\left(1/\left(2C_{2}^{\prime }\right),\delta /\left[C_{1}^{\prime }\left(1+\delta \right)\right]\right)$ satisfies these two conditions, so from now $\lambda =\lambda^{\prime *}$. We also use the following:

$$\begin{array}{c} \frac{1}{\alpha^{\prime }}=\frac{1}{\lambda^{\prime *}\left(1-\frac{\lambda^{\prime *}C_{1}^{\prime }}{2m(1-C_{2}\lambda^{\prime *}/m)}\right)}\leq \frac{\beta^{\prime }}{\lambda^{\prime *}\alpha^{\prime }}\leq \frac{\left(1+\delta \right)}{m\min(1/\left(2C_{2}^{\prime }\right),\delta /\left[C_{1}^{\prime }\left(1+\delta \right)\right])}\leq \frac{C_{1}^{\prime }{\left(1+\delta \right)}^{2}}{m\delta }. \end{array}$$

So far the bound is:

$$\begin{array}{c} P\{{∥{\hat{XMZ}}_{\lambda^{\prime *}}-XM^{*}Z∥}_{F,\Pi }^{2}\leq \underset{\bar{M}\in R^{p\times k}}{\inf}[\left(1+\delta \right){∥X\bar{M}Z-XM^{*}Z∥}_{F,\Pi }^{2}+\\ {\left(1+\delta \right)∥X∥}_{F}^{2}{∥Z∥}_{F}^{2}pk\tau^{2}+\\ \frac{C_{1}^{\prime }{\left(1+\delta \right)}^{2}\left(4\mathrm{rank}\left(\bar{M}\right)\left(k+p+2\right)\log\left(1+\frac{∥\bar{M}{∥}_{F}}{\tau \sqrt{2\mathrm{rank}(\bar{M})}}\right)+2\log\frac{2}{\varepsilon }\right)}{m\delta }]\}\geq 1-\varepsilon . \end{array}$$

In particular, with probability at least 1−ε, the choice $\tau^{2}=C_{1}^{\prime }\left(k+p\right)/{(mkp∥X∥}_{F}^{2}{∥Z∥}_{F}^{2})$ gives

$$\begin{array}{c} {∥{\hat{XMZ}}_{\lambda^{\prime *}}-XM^{*}Z∥}_{F,\Pi }^{2}\leq \underset{\bar{M}\in R^{p\times k}}{\inf}[\left(1+\delta \right){∥X\bar{M}Z-XM^{*}Z∥}_{F,\Pi }^{2}+\frac{C_{1}^{\prime }\left(1+\delta \right)\left(k+p\right)}{m}+\\ \frac{C_{1}^{\prime }{\left(1+\delta \right)}^{2}\left(4\mathrm{rank}\left(\bar{M}\right)\left(k+p+2\right)\log\left(1+\frac{{∥X∥}_{F}{∥Z∥}_{F}{∥\bar{M}∥}_{F}}{\sqrt{C_{1}^{\prime }}}\sqrt{\frac{mkp}{\left(k+p\right)\mathrm{rank}\left(\bar{M}\right)}}\right)+2\log\frac{2}{\varepsilon }\right)}{m\delta }]. \end{array}$$

   □

### Appendix A.5. Proof of Theorem 4

**Proof.** The proof is proceeded completely similar to the proof of Theorem 2, in Appendix A.3.   □

## Appendix B. Comments on Algorithm Implementation

For the case of inductive matrix completion, we write the logarithm of the density of the posterior

$$\log{\hat{\rho }}_{\lambda }\left(M\right)=-\frac{\lambda }{n}\sum_{i=1}^{n}{\left(Y_{i}-{\left(\Pi_{C}\left(XMZ\right)\right)}_{i}\right)}^{2}-\frac{p+m+2}{2}\log\det\left(\tau^{2}I_{m}+MM^{⊺}\right).$$

Let us now differentiate this expression in *M*. Note that the term ${\left(Y_{i}-{\left(\Pi_{C}\left(XMZ\right)\right)}_{i}\right)}^{2}$ does actually not depend on *M* locally if ${\left(XMZ\right)}_{i}\notin \left[-C,C\right]$, in this case its differential with respect to *M* is $0_{p\times m}$. Otherwise, ${\left(Y_{i}-{\left(\Pi_{C}\left(XMZ\right)\right)}_{i}\right)}^{2}={\left(Y_{i}-{\left(XMZ\right)}_{i}\right)}^{2}$. In order to be able to differentiate the term ${\left(XMZ\right)}_{i}$, let us introduce a notation for the entries of $I_{i}$: $I_{i}=\left(a_{i},b_{i}\right)$. Then $\nabla {\left(XMZ\right)}_{i}=D$ where the matrix $D\in R^{p\times m}$ satisfies $D_{x,y}=1_{\left\{x=b_{j}\right\}}X_{a_{j},y}$. Then

$$\nabla \log{\hat{\rho }}_{\lambda }\left(M\right)=\frac{2\lambda }{n}\sum_{i=1}^{n}\left(\nabla {\left(XMZ\right)}_{i}\right)\left(Y_{i}-{\left(XMZ\right)}_{i}\right)1_{\left\{|{\left(XMZ\right)}_{i}|<C\right\}}-\left(p+m+2\right){\left(\tau^{2}I_{m}+MM^{⊺}\right)}^{-1}M.$$

The above calculation still requires to calculate a p×p matrix inversion at each iteration; for very large *p*, this might be expensive and can slow down the algorithm. Therefore, we could replace this matrix inversion by its accurately approximation through a convex optimization. It is noted that the matrix $B:={\left(\tau^{2}I_{m}+MM^{⊺}\right)}^{-1}M$ is the solution to the following convex optimization problem: $\min_{B}\left\{∥I_{p}-M^{⊤}{B∥}_{F}^{2}+\tau^{2}{∥B∥}_{F}^{2}\right\}.$ The solution of this optimization problem can be conveniently obtained by using the package ‘glmnet’ [50] (with the family option ‘mgaussian’). This avoids performing matrix inversion or other costly calculation. However, we note here that the LMC algorithm is being used with approximate gradient evaluation; theoretical assessment of this approach can be found in [51].

**Algorithm A1** LMC
**Input**: The data. **Parameters**: Positive real numbers λ,τ,h,T. **Output**: The matrix $\hat{M}$ **Initialize**: $M_{0},\hat{M}=0_{m\times p}$ **for**k←1 to *T* **do** Sample $M_{k}$ from (8); $\hat{M}\leftarrow \hat{M}+M_{k}/T$ **end for**

**Algorithm A2** MALA
**Input**: The data. **Parameters**: Positive real numbers λ,τ,h,T **Output**: The matrix $\hat{M}$ **Initialize**: $M_{0};\hat{M}=0_{m\times p}$ **for**k=1 to *T* **do** Sample ${\tilde{M}}_{k}$ from (9). Set $M_{k}={\tilde{M}}_{k}$ with probability $A_{MALA}$, from (10), otherwise $M_{k}=M_{k-1}$. $\hat{M}\leftarrow \hat{M}+M_{k}/T$. **end for**

## References

[1] Rosen D.V. (2021). Bilinear Regression with Rank Restrictions on the Mean and Dispersion Matrix. Methodology and Applications of Statistics.

[2] Von Rosen D. (2018). Bilinear regression analysis: An Introduction.

[3] Potthoff R.F., Roy S. (1964). A generalized multivariate analysis of variance model useful especially for growth curve problems. Biometrika.

[4] Woolson R.F., Leeper J.D. (1980). Growth curve analysis of complete and incomplete longitudinal data. Commun. Stat.-Theory Methods.

[5] Kshirsagar A., Smith W. (1995). Growth Curves.

[6] Jana S. (2017). Inference for Generalized Multivariate Analysis of Variance (GMANOVA) Models and High-Dimensional Extensions. Ph.D. Thesis.

[7] Natarajan N., Dhillon I.S. (2014). Inductive matrix completion for predicting gene–disease associations. Bioinformatics.

[8] Zilber P., Nadler B. Inductive Matrix Completion: No Bad Local Minima and a Fast Algorithm. Proceedings of the 39th ICML.

[9] Zhang W., Xu H., Li X., Gao Q., Wang L. (2020). DRIMC: An improved drug repositioning approach using Bayesian inductive matrix completion. Bioinformatics.

[10] Hsieh C.J., Natarajan N., Dhillon I. PU learning for matrix completion. Proceedings of the International Conference on Machine Learning, PMLR.

[11] Jana S., Balakrishnan N., Hamid J.S. (2019). Bayesian growth curve model useful for high-dimensional longitudinal data. J. Appl. Stat..

[12] Knoblauch J., Jewson J., Damoulas T. (2022). An Optimization-centric View on Bayes’ Rule: Reviewing and Generalizing Variational Inference. J. Mach. Learn. Res..

[13] Bissiri P.G., Holmes C.C., Walker S.G. (2016). A general framework for updating belief distributions. J. R. Stat. Soc. Ser. B (Stat. Methodol.).

[14] Grünwald P., Van Ommen T. (2017). Inconsistency of Bayesian inference for misspecified linear models, and a proposal for repairing it. Bayesian Anal..

[15] McAllester D. (1998). Some PAC-Bayesian theorems. Proceedings of the Eleventh Annual Conference on Computational Learning Theory.

[16] Shawe-Taylor J., Williamson R. (1997). A PAC analysis of a Bayes estimator. Proceedings of the Tenth Annual Conference on Computational Learning Theory.

[17] Catoni O. (2007). PAC-Bayesian Supervised Classification: The Thermodynamics of Statistical Learning.

[18] Guedj B. (2019). A primer on PAC-Bayesian learning. arXiv.

[19] Alquier P. (2021). User-friendly introduction to PAC-Bayes bounds. arXiv.

[20] Mai T.T., Alquier P. (2015). A Bayesian approach for noisy matrix completion: Optimal rate under general sampling distribution. Electron. J. Statist..

[21] Cottet V., Alquier P. (2018). 1-Bit matrix completion: PAC-Bayesian analysis of a variational approximation. Mach. Learn..

[22] Mai T.T., Alquier P. (2017). Pseudo-Bayesian quantum tomography with rank-adaptation. J. Stat. Plan. Inference.

[23] Mai T.T., Alquier P. (2022). Optimal quasi-Bayesian reduced rank regression with incomplete response. arXiv.

[24] Jain P., Dhillon I.S. (2013). Provable inductive matrix completion. arXiv.

[25] Candès E.J., Plan Y. (2010). Matrix completion with noise. Proc. IEEE.

[26] Koltchinskii V., Lounici K., Tsybakov A.B. (2011). Nuclear-norm penalization and optimal rates for noisy low-rank matrix completion. Ann. Statist..

[27] Foygel R., Shamir O., Srebro N., Salakhutdinov R. Learning with the weighted trace-norm under arbitrary sampling distributions. Proceedings of the Advances in Neural Information Processing Systems.

[28] Klopp O. (2014). Noisy low-rank matrix completion with general sampling distribution. Bernoulli.

[29] Negahban S., Wainwright M.J. (2012). Restricted strong convexity and weighted matrix completion: Optimal bounds with noise. J. Mach. Learn. Res..

[30] Dalalyan A.S., Tsybakov A.B. (2012). Sparse regression learning by aggregation and Langevin Monte-Carlo. J. Comput. Syst. Sci..

[31] Dalalyan A.S. (2020). Exponential weights in multivariate regression and a low-rankness favoring prior. Annales de l’Institut Henri Poincaré Probabilités et Statistiques.

[32] Anderson T.W. (1951). Estimating linear restrictions on regression coefficients for multivariate normal distributions. Ann. Math. Stat..

[33] Izenman A.J. (2008). Modern multivariate statistical techniques. Regres. Classif. Manifold Learn..

[34] Dalalyan A., Tsybakov A.B. (2008). Aggregation by exponential weighting, sharp PAC-Bayesian bounds and sparsity. Mach. Learn..

[35] Catoni O., Picard J. (2004). Statistical learning theory and stochastic optimization. Saint-Flour Summer School on Probability Theory 2001.

[36] Alquier P., Ridgway J., Chopin N. (2016). On the properties of variational approximations of Gibbs posteriors. J. Mach. Learn. Res..

[37] Rigollet P., Tsybakov A.B. (2012). Sparse estimation by exponential weighting. Stat. Sci..

[38] Dalalyan A.S., Grappin E., Paris Q. (2018). On the exponentially weighted aggregate with the Laplace prior. Ann. Stat..

[39] Candes E.J., Wakin M.B., Boyd S.P. (2008). Enhancing sparsity by reweighted *ℓ*_1_ minimization. J. Fourier Anal. Appl..

[40] Yang L., Fang J., Duan H., Li H., Zeng B. (2018). Fast low-rank Bayesian matrix completion with hierarchical gaussian prior models. IEEE Trans. Signal Process..

[41] Luo C., Liang J., Li G., Wang F., Zhang C., Dey D.K., Chen K. (2018). Leveraging mixed and incomplete outcomes via reduced-rank modeling. J. Multivar. Anal..

[42] Hoeffding W. (1963). Probability Inequalities for Sums of Bounded Random Variables. J. Am. Stat. Assoc..

[43] Durmus A., Moulines E. (2019). High-dimensional Bayesian inference via the unadjusted Langevin algorithm. Bernoulli.

[44] Roberts G.O., Stramer O. (2002). Langevin diffusions and Metropolis-Hastings algorithms. Methodol. Comput. Appl. Probab..

[45] Roberts G.O., Rosenthal J.S. (1998). Optimal scaling of discrete approximations to Langevin diffusions. J. R. Stat. Soc. Ser. B (Stat. Methodol.).

[46] Dalalyan A.S. (2017). Theoretical guarantees for approximate sampling from smooth and log-concave densities. J. R. Stat. Soc. Ser. B (Stat. Methodol.).

[47] R Core Team (2022). R: A Language and Environment for Statistical Computing.

[48] Hastie T., Mazumder R. softImpute: Matrix Completion via Iterative Soft-Thresholded SVD, 2021. R Package Version 1.4-1. https://cran.r-project.org/package=softImpute.

[49] Massart P. (2007). Concentration Inequalities and Model Selection.

[50] Friedman J., Hastie T., Tibshirani R. (2010). Regularization Paths for Generalized Linear Models via Coordinate Descent. J. Stat. Softw..

[51] Dalalyan A.S., Karagulyan A. (2019). User-friendly guarantees for the Langevin Monte Carlo with inaccurate gradient. Stoch. Process. Their Appl..

